# Supplementary material for: A dark intermediate in the fluorogenic reaction between tetrazine fluorophores and trans-cyclooctene
Source: Biophys Rep (N Y). 2022 Nov 5;2(4):100084. doi: 10.1016/j.bpr.2022.100084 (PMC9782730; doi:10.1016/j.bpr.2022.100084)
Supplement: Document S1. Figures S1–S8 [file mmc1.pdf]

Biophysical Reports, Volume *2*

Supplemental information

A dark intermediate in the fluorogenic reaction between tetrazine fluorophores and *trans*-cyclooctene

Felix Hild, Philipp Werther, Klaus Yserentant, Richard Wombacher, and Dirk-Peter Herten

Supplemental Information

A dark intermediate in the fluorogenic reaction between tetrazine fluorophores and trans-cyclooctene

Felix Hild, Philipp Werther*, Klaus Yserentant, Richard Wombacher, Dirk-Peter Herten*

* These authors contributed equally to the manuscript.

Figure S1

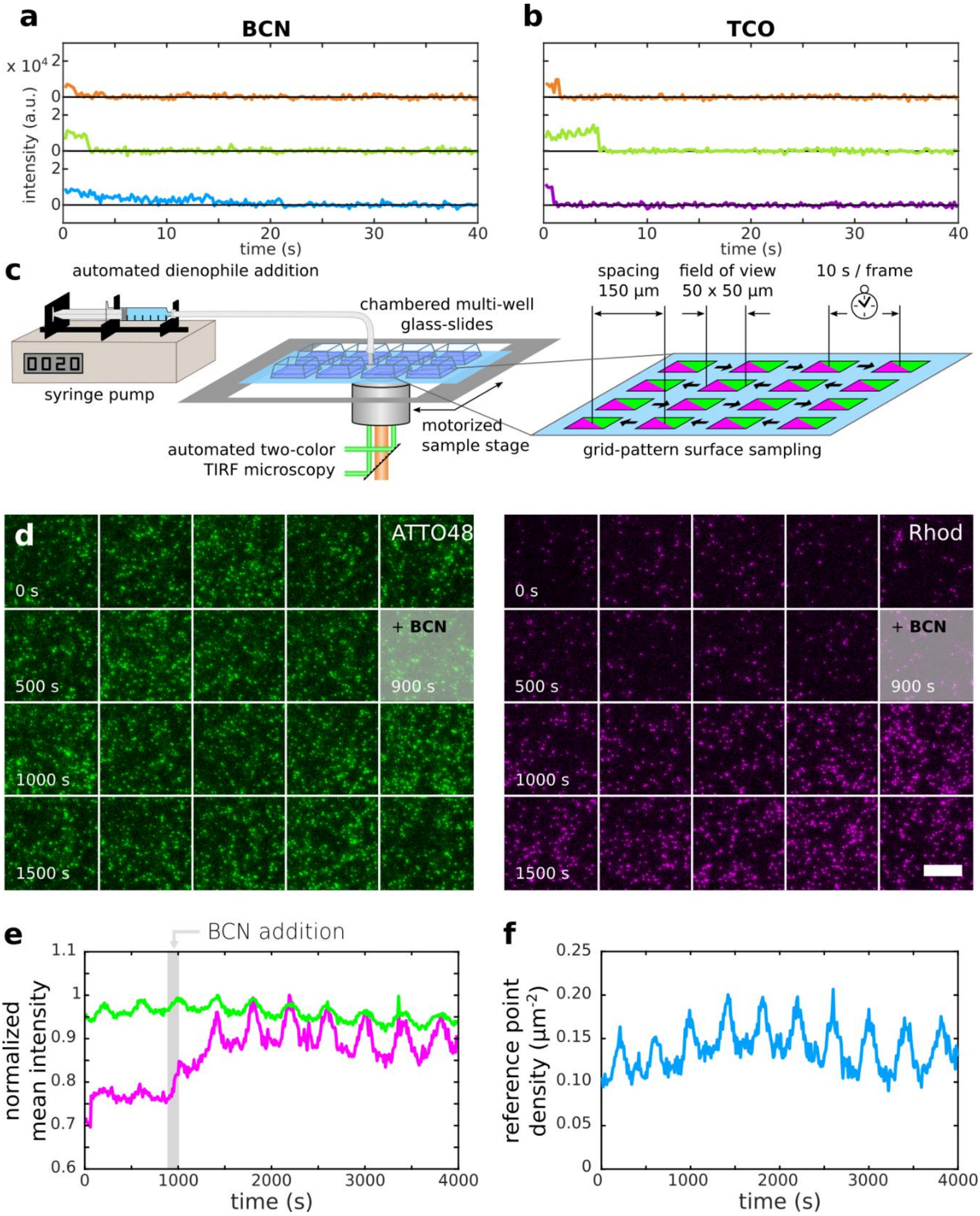

Figure S1: Examples of single molecule intensity traces of IEDDA reaction products of immobilised Rhod-Tz reacted with 50 µM a) BCN and b) TCO. The rapid photo-bleaching of these products required a different concept of time-resolved subpopulation probing explained in the following. c) Automated two-colour time-lapse single-molecule microscopy of fluorogenic IEDDA reaction was realized with automated dienophile addition with a motorized syringe pump, a motorized stage moving chambered glass slides and an automated multi-colour microscopy acquisition. To sample the progress of the reaction the glass surface was scanned at 10 s / frame in a serpentine pattern with 150 µm spacing. d) Microscopy images show an increase in point density in the Rhod channel (magenta) after BCN addition at nearly constant density in the ATTO488 reference channel (green). e) Normalized mean image intensity profiles show an increase after addition in the magenta channel with superimposed periodic fluctuations in both channels due to variations in reference point density (f). Scale bar 10 µm. Snapshots of acquired images show a roughly constant point density for ATTO488 in (d). In contrast, the point density of Rhod-Tz increases significantly after addition of BCN at 900 s. This effect is clearly visible in a plot of mean image intensity normalized to the maximum of the sequence (e). Additionally, it shows periodic fluctuations correlating between the two spectral channels. These variations occurring with a spacing of 20 frames are due to a variation of template density over the scanned region which controls the achievable density of Rhod and is reflected in the mean intensity of the images. This is evident with a plot of the density of points detected in theATTO488 channel over time in (f). Such large-scale inhomogeneities are frequently observed for fluorophores immobilized by this concept and could be a result of uneven surface quality of the glass or non-uniform treatment during sample preparation. Generally, this should not impose a problem as the observed densities of 0.1–0.2 µm-2 correspond to 250–500 points per field of view and herein provides sufficient data for statistically relevant analysis.

Figure S2

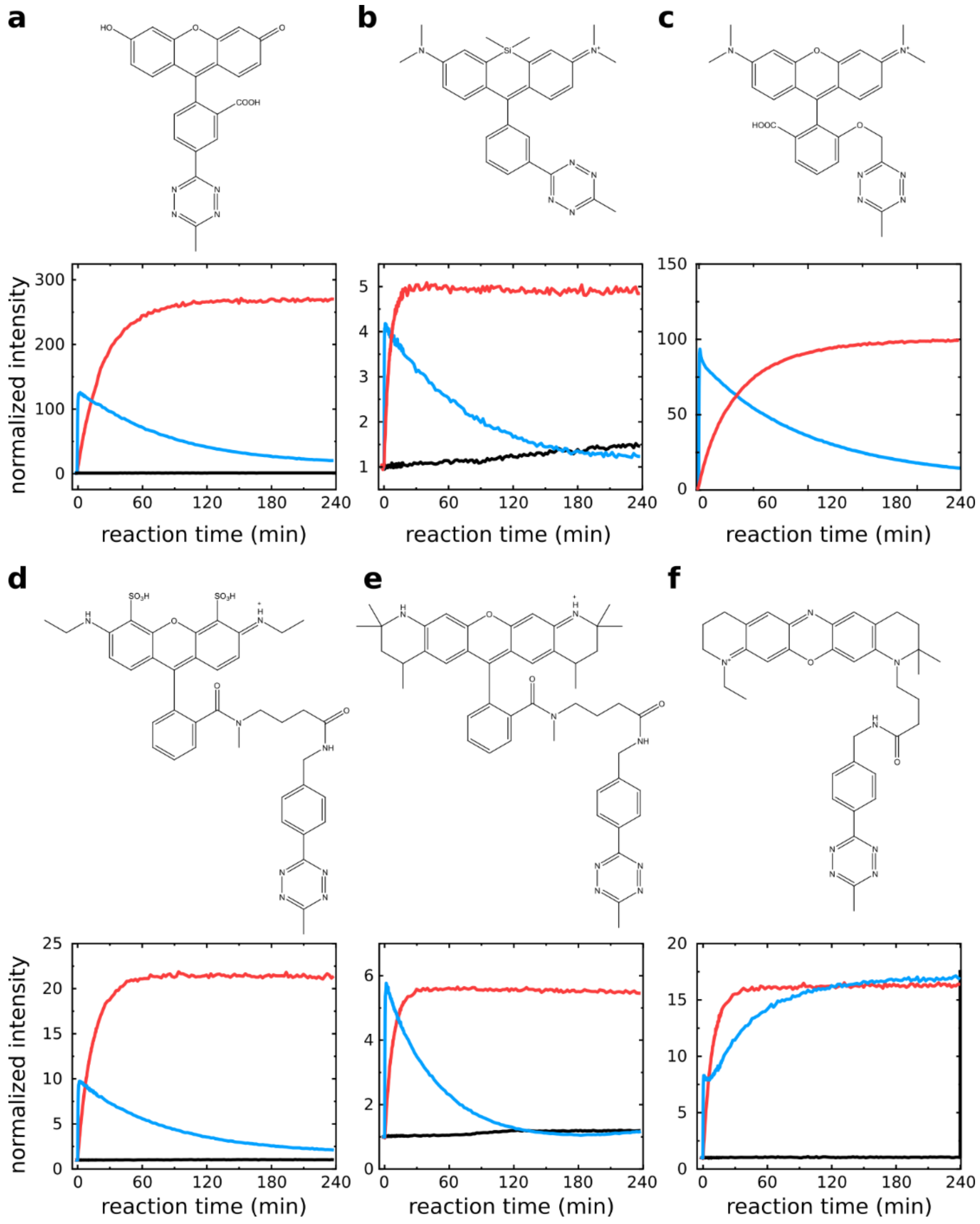

Figure S2: Fluorescence time course measurements of fluorogenic Tz dyes. a) Fl-Tz, b) SiR-Tz, c) TMR-Tz, d) ATTO532-Tz, e) ATTO550-Tz and f) ATTO655-Tz, all 5 µM in PBS, reacting with a 20-fold excess of BCN (red) or TCO (blue). Fluorescence intensity was recorded over 4 h at the wavelengths of the individual emission maxima. All dyes reacted with TCO except ATTO655-Tz show a significant drop in intensity after about 5 mins. ATTO655-Tz reproducibly shows a steep increase with only a short and small decrease to an intermediate level within 5 mins followed by an increase over 2 h saturating at an intensity that is comparable to the reaction with BCN. This is most likely due to a faster oxidation to the final pyridazine. Control samples (black) show neglectable increase for a), b), d), e) and f) and a higher instability for c).

Figure S3

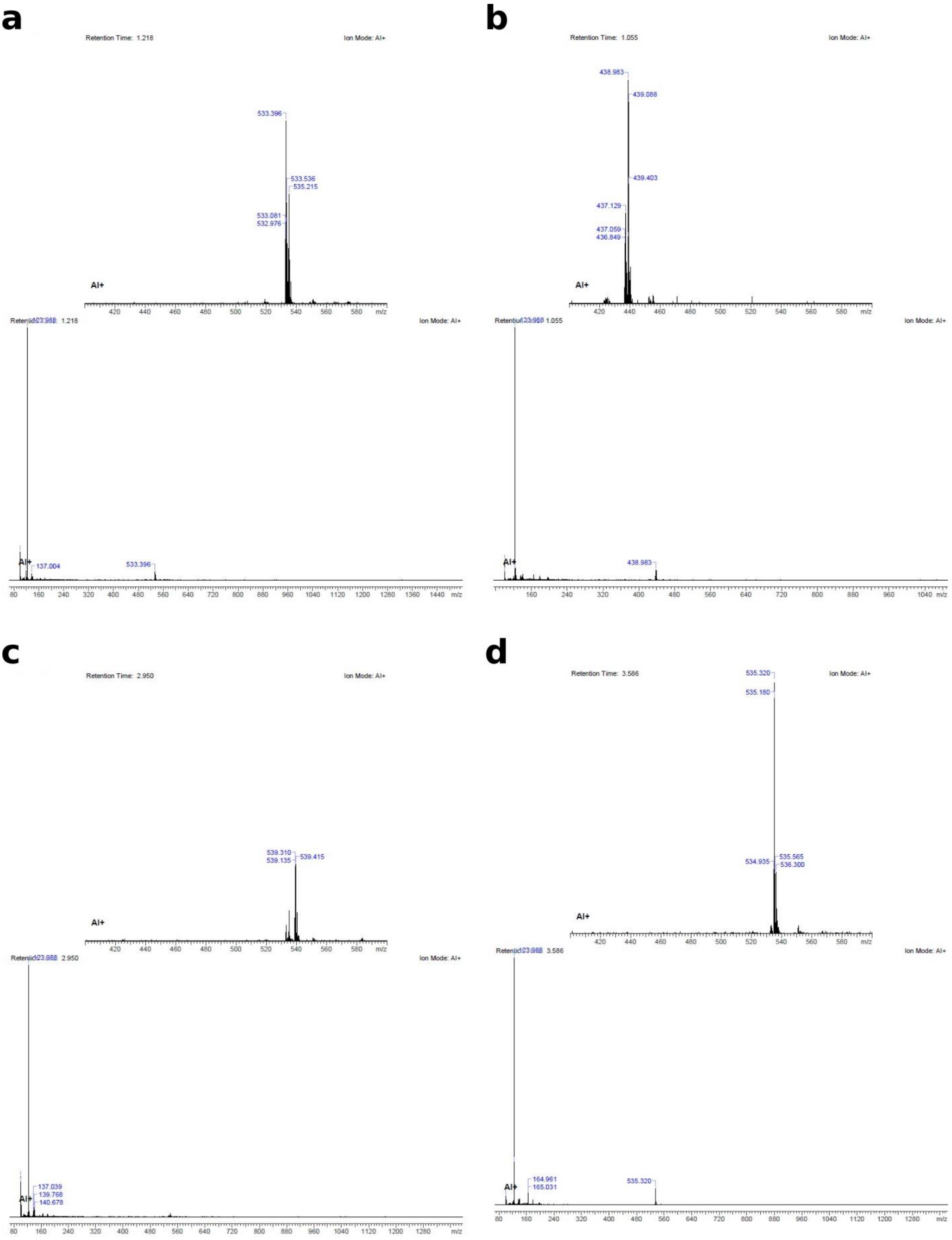

Figure S3: Mass spectra of relevant peaks from the ULPC-MS experiments in fig. 1b. a) Peak I at t = 14d, b) peak II at t = 8d, c) peak IV at t = 5 min and d) peak V at t = 8h.

Figure S4

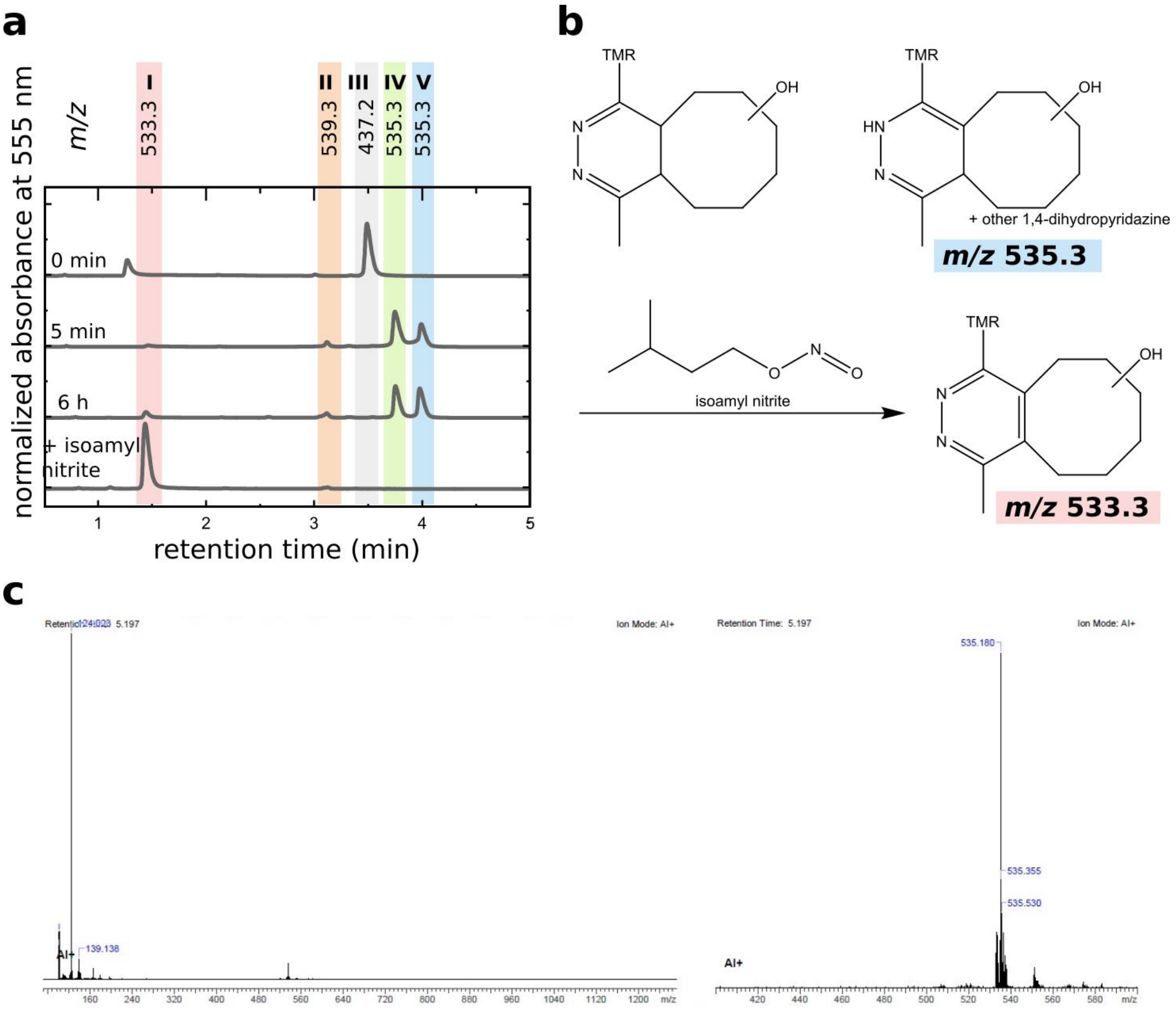

Figure S4: a) UPLC-MS analysis of the b) oxidation of dihydropyridazines with isoamyl nitrite. 4,5- and 1,4-dihydropyridazines (peaks IV and V) were oxidized by addition of 100 eq of isoamyl nitrite yielding a single peak with m/z 533.3 corresponding to the pyridazine (peak I). c) Mass spectrum of peak I after oxidation with isoamylnitride.

Figure S5

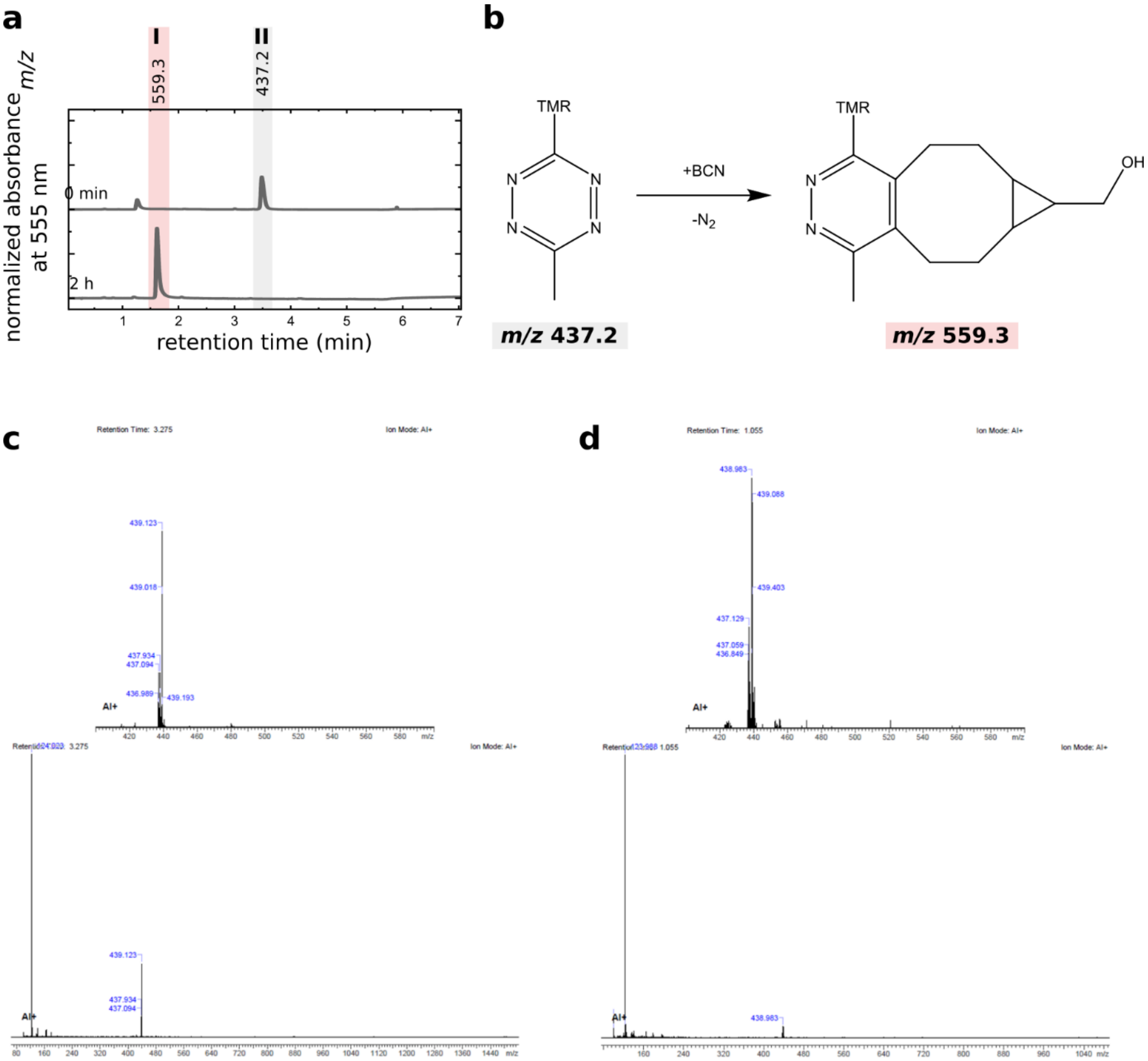

Figure S5: UPLC-MS analysis of TMR-Tz reacting with BCN. a) Chromatograms of the b) reaction of TMR-Tz (m/z 437.2) at 200 µM in PBS before (0 min) and 2 h after addition of 20 equivalents of BCN to form a single pyridazine product (m/z 559.3). c) Mass spectra of peak II at t = 0 and d) peak I at t = 2h.

Figure S6

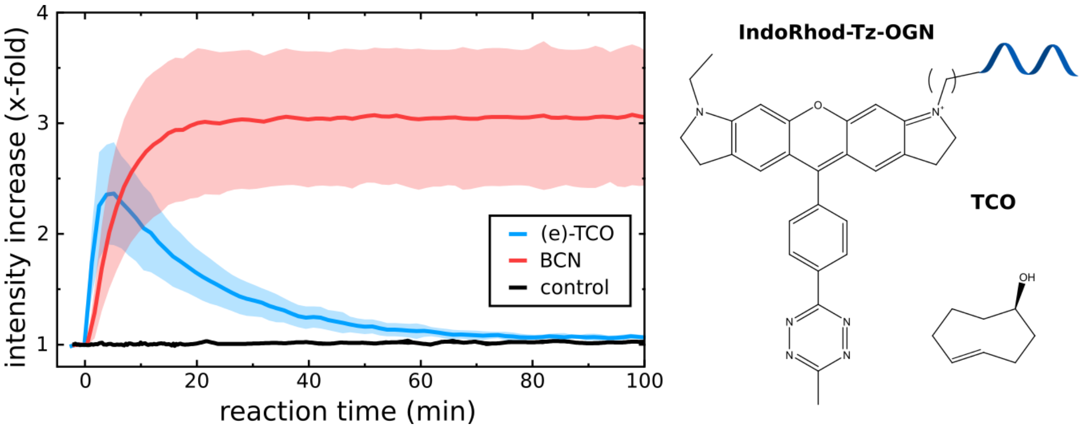

Figure S6: Reaction of the oligonucleotide coupled IndoRhod-Tz with dienophiles. Time course measurements of fluorescence intensity at λ_{em} 595 nm excited at λ_{ecx} 535 nm. Reaction with TCO (blue) yields a steep increase followed by a decrease nearly down to the initial intensity level. BCN (red) shows a slower increase to a constant level. Control samples (black) without dienophile addition. Lines represent the average of three measurements with the standard deviation shown as transparent bands.

Figure S7

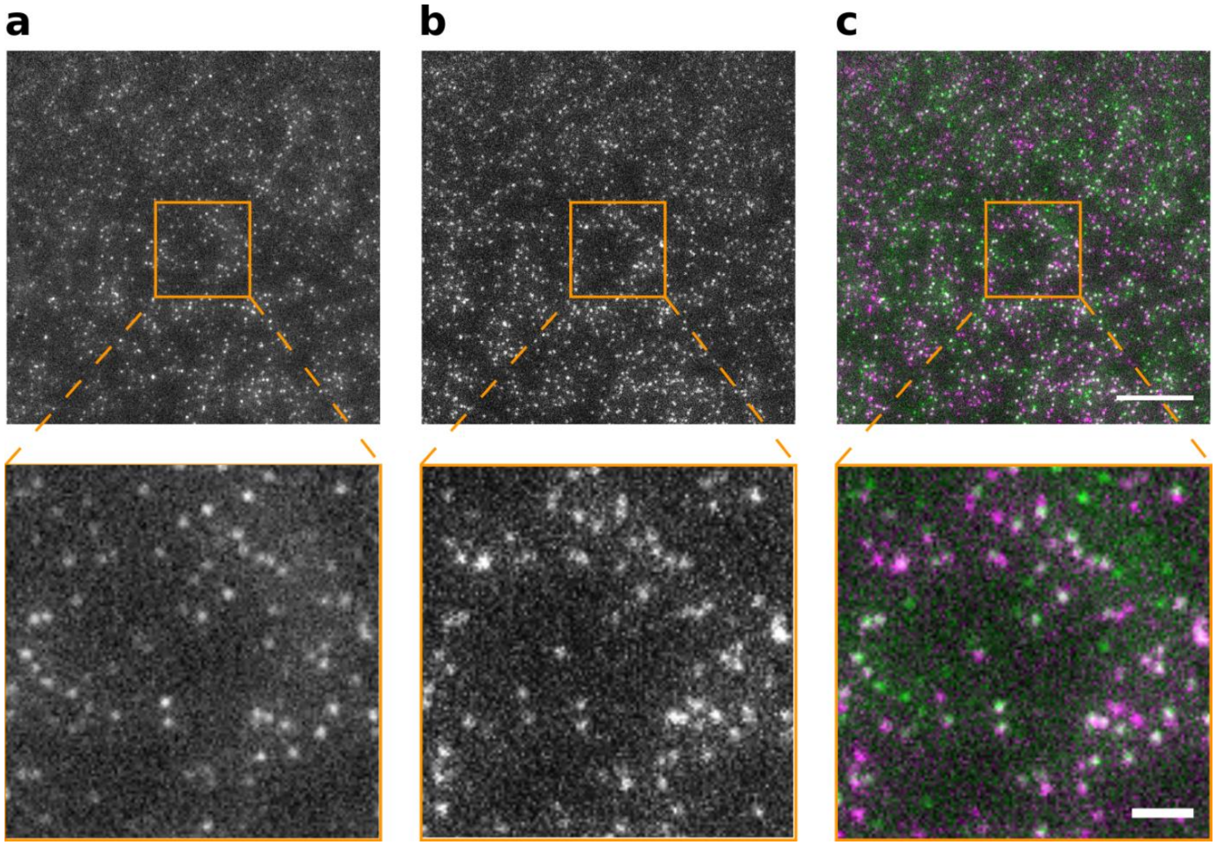

Figure S7: Two-colour TIRF microscopy of immobilized Rhod-Tz after reaction with BCN. The template oligonucleotide labelled with ATTO488 (green) and Rhod-Tz after reaction with BCN (magenta) shows a high degree of colocalization (white points in overlay). Scale bar 10 µm (top) and 2 µm (bottom).

Figure S8

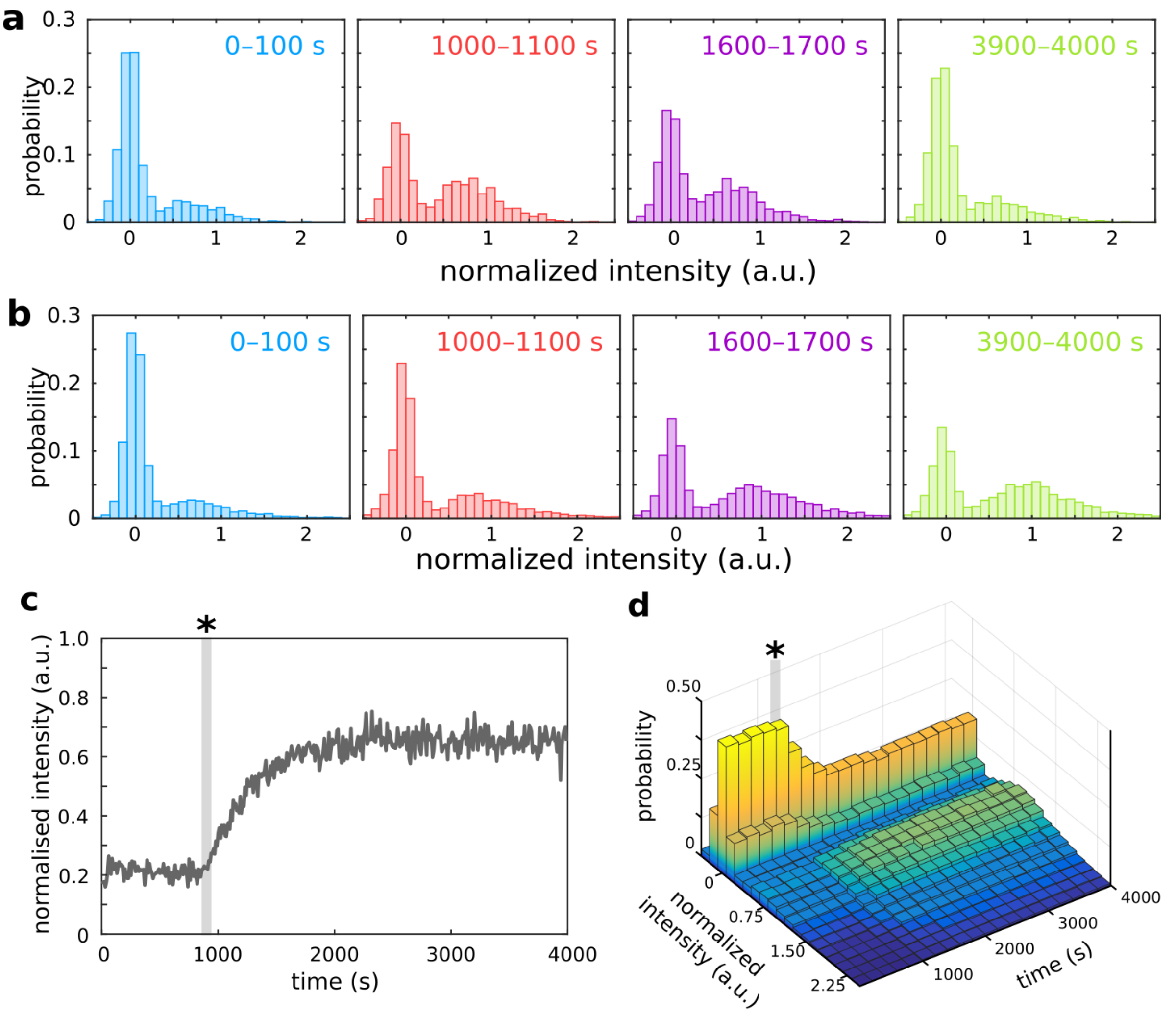

Figure S8: Time-resolved single molecule studies of the reaction of Rhod-Tz with different dienophiles. a) Intensity distributions after 100, 1100, 1700 and 4000 s show the formation of a bright population briefly after TCO addition at 900 s while the background population simultaneously decreases. Over time the trend is reversed and results in a distribution after ca. 4000 s that approximately corresponds to the initial distribution. b) Intensity histograms after 100, 1100, 1700 and 4000 s show a steady increase of a bright population after addition of BCN (added at 900 s) while the background intensity population decreases. c) Ensemble intensity time course of the reaction of Rhod-Tz after addition of BCN (marked with *) from averaging intensities per frame (grey, top) shows the corresponding overall intensity. d) The 3D-visualization of background corrected and normalized intensity distributions upon addition of BCN (marked with *) plotted against time shows a continuous growth of the bright population along with a simultaneous decrease of the dark population saturating about 1000 s after BCN addition.
