# Supplementary material for: A dark intermediate in the fluorogenic reaction between tetrazine fluorophores and trans-cyclooctene
Source: Biophys Rep (N Y). 2022 Nov 5;2(4):100084. doi: 10.1016/j.bpr.2022.100084 (PMC9782730; doi:10.1016/j.bpr.2022.100084)
Supplement: Document S2. Article plus supporting material [file mmc2.pdf]

Biophysical Reports

ARTICLE

A dark intermediate in the fluorogenic reaction between tetrazine fluorophores and *trans*-cyclooctene

Felix Hild,[1] Philipp Werther,[2] Klaus Yserentant,[1,3,4] Richard Wombacher,[2,5] and Dirk-Peter Herten[1,3,4,*]
[1]Physikalisch-Chemisches Institut, Heidelberg University, Heidelberg, Germany; [2]Institute of Pharmacy and Molecular Biotechnology, Heidelberg University, Heidelberg, Germany; [3]Institute of Cardiovascular Sciences, College of Medical and Dental Sciences and School of Chemistry, University of Birmingham, Birmingham, United Kingdom; [4]Centre of Membrane Proteins and Receptors (COMPARE), The Universities of Birmingham and Nottingham, Birmingham, West Midlands, United Kingdom; and [5]Max-Planck-Institut für Medizinische Forschung, Heidelberg, Germany

ABSTRACT Fluorogenic labeling via bioorthogonal tetrazine chemistry has proven to be highly successful in fluorescence microscopy of living cells. To date, *trans*-cyclooctene (TCO) and bicyclonyne have been found to be the most useful substrates for live-cell labeling owing to their fast labeling kinetics, high biocompatibility, and bioorthogonality. Recent kinetic studies of fluorogenic click reactions with TCO derivatives showed a transient fluorogenic effect but could not explain the reaction sequence and the contributions of different intermediates. More recently, fluorescence quenching by potential intermediates has been investigated, suggesting their occurrence in the reaction sequence. However, in situ studies of the click reaction that directly relate these observations to the known reaction sequence are still missing. In this study, we developed a single-molecule fluorescence detection framework to investigate fluorogenic click reactions. In combination with data from ultra-performance liquid chromatography-tandem mass spectrometry, this explains the transient intensity increase by relating fluorescent intermediates to the known reaction sequence of TCO with fluorogenic tetrazine dyes. More specifically, we confirm that the reaction of TCO with tetrazine rapidly forms a fluorescent 4,5-dihydropyridazine species that slowly tautomerizes to a weakly fluorescent 1,4-dihydropyridazine, explaining the observed drop in fluorescence intensity. On a much slower timescale of hours/days, the fluorescence intensity may be recovered by oxidation of the intermediate to a pyridazine. Our findings are of importance for quantitative applications in fluorescence microscopy and spectroscopy as the achieved peak intensity with TCO depends on the specific experimental settings. They clearly indicate the requirement for more robust benchmarking of click reactions with tetrazine dyes and the need for alternative dienophiles with fast reaction kinetics and stable fluorescence emission to further applications in advanced fluorescence microscopy.

WHY IT MATTERS Bioorthogonal click chemistry has enabled alternative routes for fluorescence labeling of biomolecules in living cells. In particular, Diels-Alder reactions with inverse electron demand of tetrazine dyes with *trans*-cyclooctene or bicyclononyne functionalized substrates have gained strong interest as they allow fluorescence labeling in living cells without washing due to their fluorescence uncaging properties. In contrast to bicyclononyne, the labeling quality of *trans*-cyclooctene is somewhat variable where usually an initial peak intensity is observed, which levels off over several minutes to hours. In this study, we combined single-molecule fluorescence imaging with ultra-performance liquid chromatography-tandem mass spectrometry to identify the reaction intermediates explaining the dynamic changes in fluorescence intensity over the course of the click reaction. Our investigations are of importance for the general understanding of fluorogenic tetrazines and guide the use of dienophiles in bioorthogonal tetrazine labeling schemes.

INTRODUCTION

Recent developments in bioorthogonal labeling have opened promising perspectives for a broad range of applications in fluorescence spectroscopy and microscopy (1–8). Especially, single-molecule fluorescence and superresolution microscopy benefit substantially from the possibility of using organic fluorophores that

Submitted August 18, 2022, and accepted for publication November 2, 2022.
*Correspondence: d.herten@bham.ac.uk
Felix Hild and Philipp Werther contributed equally to this work.
Editor: Jorg Enderlein.
https://doi.org/10.1016/j.bpr.2022.100084
© 2022 The Authors.
This is an open access article under the CC BY license (http://creativecommons.org/licenses/by/4.0/).

fulfill specific experimental needs, such as higher brightness and photostability (9). Bioorthogonal labeling also enables further minimization of the label size, which is highly relevant with regards to the increase in optical resolution in superresolution microscopy as well as to biocompatibility of labeling experiments in living cells (10).

Among other bioorthogonal reactions, the Diels-Alder reaction with inverse electron demand between tetrazines and strained alkenes and alkynes stands out due to its fast kinetics and biocompatibility. The orthogonality to reactions and compounds in living systems qualifies this technology for effective cellular labeling (11–14) and bioconjugation (15).

In kinetic studies, *trans*-cyclooctene (TCO) and bicyclononyne (BCN) have been identified as dienophile structures for fast and reliable labeling without compromising biocompatibility (14,16,17). So far, derivatives thereof are the only dienophiles of practical use for live-cell imaging at low concentrations, negligible side reactions, and reasonable incubation times. Since then, the field has moved to exploit the fluorescence quenching properties of tetrazine (12,18) for the development of fluorogenic probes. These facilitate sample preparation by reducing the need for extensive washing of unbound fluorophore and enable no-wash labeling (16,19–22). This has been shown to be highly beneficial for a range of applications including superresolution microscopy (17,23–25).

For a maximum fluorogenic effect, the structural development of tetrazine-dye conjugates aims at achieving high quenching efficiencies for a pronounced intensity increase due to the labeling reaction (turn-on ratio) (17,18). Nevertheless, such dyes have to fulfill general demands for biocompatibility, live-cell labeling, and fluorescence microscopy. Here, red-shifted fluorophores were found to be advantageous due to reduced impact of autofluorescence and a lower energy input in the sample, which preserves cell physiology and increased penetration depths (22).

Despite their successful application in a range of areas, the intensity variations observed for reactions with TCO are still not well understood. For example, in recent kinetic studies of fluorogenic click reactions with TCO derivatives, we and others found that the fluorescence intensities peaked after a few minutes, followed by a continuous decay of intensity over several hours (Fig. 1 *a*) (14,16,22). This is in clear contrast to the reaction with BCN, which showed a slower but steady increase in fluorescence that goes into saturation at intensities similar to the maximum intensity achieved with TCO derivatives. We also observed that a tetrazine-functionalized fluorogenic rhodamine (TMR-Tz) showed a lower turn on in live-cell experiments upon reaction with HaloTag functionalized with *trans*-cyclooct-2-en-1-ol compared with BCN (22). Similar observations have initiated investigations of the quenching properties of the different reaction products of a tetrazine derivative with TCO and BCN. The authors conclude that the dihydropyridazine derivative resulting from the reaction with TCO may contribute to significant fluorescence quenching, while the pyridazine resulting from reactions with BCN shows almost no quenching (26). While these studies indicate potential intermediates that might quench fluorescence emission, the order of their occurrence and their contribution to the apparent fluorescence emission remain obscure. Here, simultaneous analytics of photophysical properties and assignment of molecular structures to transient species in homogeneous solution remain challenging. This leaves uncertainties for quantitative applications in fluorescence microscopy. Clearly, a better understanding of the reaction mechanism will not only help develop better dyes for click-chemistry-based labeling in cells but, moreover, will improve the development of dienophiles to achieve more robust labeling yields.

Over the past decade, we and others have explored the potential of single-molecule fluorescence spectroscopy to study the mechanisms of chemical reactions in homogenous systems (27,28). For this study, we therefore chose time-resolved single-molecule fluorescence imaging to track the evolution of different fluorescent subpopulations occurring in the Diels-Alder reaction with inverse electron demand reactions of tetrazine dyes with *trans*-cyclooct-2-en-1-ol or BCN over the timescale of minutes to hours. Additionally, we experimentally identified different reaction intermediates over the timescale of several minutes to days by ultra-performance liquid chromatography-tandem mass spectrometry (UPLC-MS). Furthermore, we present the correlation of these data sets with bulk experiments and its comparison with the known reaction mechanism before discussing relevant consequences for use of the different dienophiles in fluorescence microscopy.

MATERIALS AND METHODS

Fluorescence spectroscopy

Time course fluorescence measurements for the study of fluorogenic reactions between tetrazine-substituted fluorophores and dienophiles were recorded on Cary Eclipse at 25°C in black quartz glass cuvettes (105.251-QS, Hellma Analytics, Müllheim, Germany, minimum volume 34 μL) using the manufacturer's Kinetics software. Oligonucleotide-coupled tetramethylrhodamine tetrazine (TMR-Tz) was diluted to 5 μM concentrations in phosphate-buffered saline (PBS), and the dienophile was added before data acquisition by adding a small volume of 20 equivalents dienophile in PBS. To minimize fluorophore dilution, the added volume was always kept below 2% of the sample volume. The cuvettes were sealed with rubber plugs to avoid

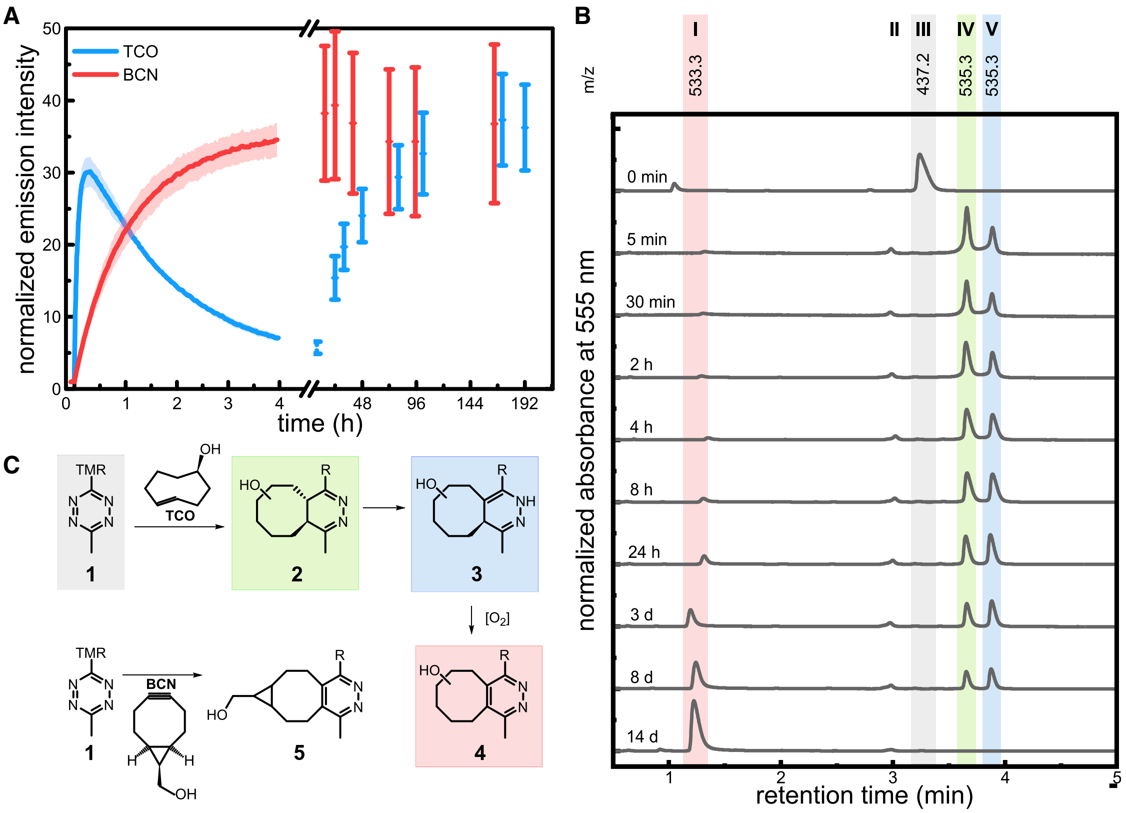

FIGURE 1 (*A*) Fluorescence transients of the reactions of TMR-Tz **1** (5 μM in PBS) with 20 equivalents of TCO (blue) and BCN (red) recorded for up to 8 days ($\lambda_{exc} = 520$ nm; $\lambda_{em} = 585$ nm). Intensity was normalized to the intensity before dienophile addition. (*B*) Time course reaction analysis of TCO with TMR-Tz **1** recorded with UPLC-MS. Color labels assign the measured *m/z* of expected intermediates according to the known reaction sequence shown in (*C*) (Fig. S3). (*C*) Schemes of the reaction of TMR-Tz **1** with TCO to 4,5-dihydropyridazine **2**, tautomerization to 1,4-dihydropyridazine **3**, and subsequent oxidation to pyridazine **4** in contrast to the direct reaction of TMR-Tz **1** with BCN to the respective pyridazine **5**.

solvent evaporation. Samples were excited at 520 nm using an excitation slit of 10 nm, and emission was recorded at 585 nm with an emission slit of 10 nm using a detector voltage of 530 V. The fluorescence intensity was normalized to the intensity before dienophile addition.

Chromatography

Analytical HPLC was carried out on an HPLC instrument (HP Series 1100, Agilent, Santa Clara, CA) equipped with an inverse phase column (C18, Hypersil ODS, 5 μm particle size, Säulentechnik Knauer, Berlin, Germany). Mobile phase buffer A (10 mM triethyl ammonium acetate [TEAA] in water) and B (10 mM TEAA in 75% iso-propanol and 25% water) were used in a gradient of 100% to 0% A over 30 min and a flow rate of 3 mL/min unless stated otherwise. Preparative HPLC for purification of oligonucleotides was performed on an inversed phase column (C18, 5 μm particle size, Phenomenex Luna, Torrance, CA, USA) using a gradient from buffer A (100 mM TEAA in water) to buffer B (100 mM TEAA in 80% acetonitrile and 20% water).

Analytical UPLC-MS detection was performed on a UPLC instrument (Acquity, Waters, Milford, MA, USA)-equipped photodiode detector and a single quadrupole mass analyzer with electrospray ionization and atmospheric pressure chemical ionization combination ion source (SQD2, Waters). Unless stated otherwise, an inverse phase column (C18, BEH, 1.7 μm particle size, Acquity UPLC, Waters) was used with mobile phase consisting of buffers A (0.1% formic acid in acetonitrile) and B (0.1% formic acid and 0.5% ammonia in water) and an eluant gradient from 30% to 50% A over 5 min with a flow rate of 0.6 mL/min. Fluorophore products were detected at an absorption wavelength of 555 nm and analyzed by MS with atmospheric pressure chemical ionization in positive mode. For UPLC-MS analysis of reactions between TMR-Tz and different dienophiles, the dye was diluted at 200 μM in PBS and reacted with a 20-fold excess of dienophile. Analytic UPLC-MS runs were sequentially performed by injection of 2 μL of the reaction mix that was kept at 4°C over the full course of the experiment. Assuming a constant extinction of involved components, absorption chromatograms at 555 nm were normalized to the summed intensity. Assignment of peaks in the absorption chromatogram were made using mass spectrograms. UPLC-MS chromatograms and spectra were processed using Spectrus Processor (v.2019.1.1, ACD/Labs, Toronto, ON, Canada).

Immobilization of the single-molecule probe

For immobilization of biotin-coupled DNA on the glass surface, LabTek (Nunc) wells were cleaned by incubation with 100 mM aqueous hydrofluoric acid for 1 min followed by three washing steps with PBS. Then, a mixture of BSA and biotin-labeled BSA (BSA/biotin-BSA 1:8, 5 mg/mL in PBS) was incubated for 1 h at room temperature to block the glass surface against unspecific adsorption and to deposit specific binding sites. After washing three times with PBS, a solution of streptavidin (100 μg/mL) was incubated for 20 min, followed

by another three washing steps with PBS and incubation with the biotin-coupled compound at nanomolar concentrations.

The rhodamine Tz (Rhod-Tz) NHS ester was coupled to a terminal amine of a 14-mer oligonucleotide (5′-TCTTACGCCAACGA-3′) as described previously (29). For single-molecule immobilization, it was hybridized at 10-fold excess (100 nM) with the complementary biotin-coupled template oligonucleotide (10 nM, ATTO488-5′-AGG CAA GCA CTT CAT CTG TTG GCG TAA GA-3′-Biotin-TEG) in PBS for 20 min at room temperature, diluted 1:2,000 in PBS, and incubated on streptavidin-covered surfaces for 10 min, followed by three washing steps with PBS.

Automated single-molecule fluorescence microscopy

Single-molecule fluorescence microscopy was performed on a custom epi-/total internal reflection fluorescence (TIRF) microscope assembled around an inverted microscope (Ti-E, Nikon, Tokyo, Japan) equipped with a halogen lamp for bright-field illumination and a multiwavelength laser light source for fluorescence excitation. The multilaser engine (iChrome MLE-LFA, Toptica, Pittsford, NY, USA) housed three diode lasers emitting at 405, 488, and 640 nm and a diode-pumped solid-state laser emitting at 561 nm that were fiber coupled and mounted to the microscopy body with a micro attenuator switch between epi and TIRF illumination. The laser beam was reflected by a quad-band dichroic filter (Di01-R405/488/561/635, Semrock, West Henrietta, NY, USA) into the sample through an oil-immersion objective (CFI Apo TIRF 100× oil, 1.49 numerical aperture, Nikon, combined with a 1.5× tube lens).

Light from the sample was collected by the objective and directed through a notch filter (TIRF-Quad filter set 405/488/561/640, AHF Analysentechnik, Tübingen, Germany) and a motorized filter wheel (FW102C, Thorlabs, Jessup, MD, USA). Fluorescence emission was filtered by bandpass filters (525/25 ET and 605/70 ET, AHF Analysentechnik). Emission light was detected with an electron multiplying charge-coupled device camera (iXon Ultra 897, Andor, Belfast, UK). To minimize redundant sample illumination, the laser source was synchronized with the camera TTL output indicating exposure. A microcontroller board (Arduino Uno, Arduino Srl, Monza, Italy) was used to route the signal to the four individual laser channels. Samples were mounted on a motorized XY stage (Märzhäuser, Wetzlar, Germany) and focused with a motorized nosepiece equipped with an auto-focus system (PFS, Nikon). For automated addition of liquids during microscopy experiments, a syringe was installed onto a syringe pump (Aladdin AL1000, World Precision Instruments, Sarasota County, FL, USA) and extended with a tube-mounted needle that was placed above the sample (addition rate of 1 mL/min). Generally, multichannel microscopy was performed with the following combinations of light source and bandpass filter: 488 nm laser excitation with 500–550 nm bandpass filter (ATTO488) and 561 nm laser excitation with 570–640 nm bandpass filter (Rhod-Tz). The microscope was controlled by open-source microscopy software Micro-Manager (v.1.4) (30,31) and operated through the graphical user interface and custom Beanshell scripts. Image processing and analysis was performed with ImageJ-based software package Fiji (v.2.0.0, ImageJ v.1.52) (32). Data processing, analysis, and plotting were performed in Matlab (v.R2016b, MathWorks, Natick, MA, USA) and Origin (v.2016G, OriginLab, Northampton, MA, USA).

Automated single-molecule time course imaging

Sequential subpopulation probing of immobilized Rhod-Tz during the reaction with dienophiles was performed by automated two-color TIRF microscopy of multiple sample positions at defined time points (Fig. S1). Multiwell glass slides were used as a sample container to generate replicates without removing the sample, allowing sequential experimental replicates with high reproducibility. Hardware components for two-color imaging, sample positioning, and dienophile addition were implemented in a universal open-source microscopy software (30,31). Essentially, excitation lasers, camera, motorized filter wheel, sample stage, and a syringe pump were synchronized with custom-written scripts to sample the surface in a predefined pattern. The sample was positioned in the corner of a LabTek well, and the surface was focused. Then, a grid of 20 × 20 positions was sequentially imaged at a rate of 6 positions/min with a spacing of 200 μm (twice the field of view) to prevent premature bleaching. This represents an area of roughly 3 × 3 mm that was manually targeted in the center of an 8 × 8 mm well. At every position, first Rhod-Tz was imaged (500 ms exposure time, 0.6 mW laser intensity, emGain 200), followed by the reference dye ATTO488 (500 ms exposure time, 3.5 mW laser intensity, emGain 200). After 15 min (90 positions), a solution of the dienophile in PBS was added automatically with a syringe pump to yield a concentration of 50 μM. The temporal resolution was limited by the hardware to 10 s per field of view, yielding 400 time points with 200–500 spots in each channel within an observation time of 67 min.

For image analysis, automated point detection was performed in both channels after intensity flat fielding to account for the irradiation laser beam profile. Channel mapping was performed in the first frame containing more than 50 pairs of nearest neighbors with a distance below 2 pixels. Single-molecule fluorescence intensities were extracted from flat-fielded images by aperture photometry and corrected for background emission. Briefly, the intensities of individual molecules were calculated via aperture photometry using an aperture (radius 3 pixel) centered on the pixel in the spot showing highest intensity and a ring of 3 pixel width with a spacing of 2 pixel from the aperture edge to determine the background signal (33). Subsequently, positions detected in the reference dye channel were mapped to the Rhod channel, and average intensities along with the local background were calculated. To account for local inhomogeneities and variations in excitation power due to different TIRF illumination angle settings, Rhod intensities were normalized to reference dye intensities.

RESULTS

Our study started with kinetic measurements of the reaction of the fluorogenic dye TMR-Tz **1** (Fig. S2 *c*) with different dienophiles in the bulk (Fig. 1 *a*). We monitored the fluorescence intensity during its reaction with TCO and BCN over a total of 8 days. The reaction with BCN leads to a steady rise in the fluorescence emission saturating after about 4 h at ca. 34-fold intensity increase and remained constant until the end of the experiments. In contrast to this, TCO, owing to its fast reaction kinetics, reaches a maximum at ca. 30-fold intensity increase already after about 20 min, followed by a significant intensity drop over the next 4 h. Only on the timescale of several days does the fluorescence intensity recover, saturating at levels comparable to those observed in the reaction with BCN. Similar observations were made for a variety of different fluorophores including different fluorophore scaffolds as well as different types of linkage to the Tz group (Fig. S2).

We then used UPLC-MS to identify the different intermediates of the reaction of **1** with TCO and their occurrence over time (Fig. 1 *b*) based on the expected masses according to the known reaction sequence (Figs. 1 *c* and S3) (34). Sequential analysis of the reaction mixture of **1** (m/z 437.2, peak **III**) with a 20-fold excess of TCO shows a rapid formation of dihydropyridazine **2**, which tautomerizes to **3** and other isomers (*m/z* 535.3, peaks **IV** and **V**). The delayed emergence of peak **V** allows attribution to the 1,4-dihydropyridazine **3**, in accordance with recent findings by Pinto-Pacheco et al. that 1,4-dihydropyridazines can significantly quench different fluorophores (26). The pyridazine **4** (*m/z* 533.3, peak **I**) emerges to the single main product peak only after several days. Apparently, this last step is an oxidation of **3** and **4** most likely by dissolved molecular oxygen. This was confirmed in additional experiments where the products formed under oxidizing conditions in the presence of isoamyl nitrite have the same *m/z* and retention time (Fig. S4). Again, the observed increase in fluorescence emission is in good agreement with the finding that pyridazines only weakly quench fluorescence emission (26). The remaining peak (*m/z* 438.9, peak **II**) formed early in the reaction could be attributed to a pyridazine hydrolysis product or an isomeric structure (Fig. S3 *b*). Comparable experiments with BCN as dienophile show the direct formation of the respective pyridazine **5**, as suggested by the known reaction mechanism (Figs. 1 *c* and S5).

While the UPLC-MS experiments give a good indication for the order of occurrence of the different intermediates, it remained unclear whether and to what extent both dihydropyridazines **2** and **3** would contribute to the fluorescence emission of the reaction product with TCO. We sought to address this by time-resolved single-molecule fluorescence studies to establish an experimental framework enabling in situ observation of transiently occurring species during the click reaction. We used a previously published bifunctional fluorogenic rhodamine probe attached to a 14-mer DNA oligonucleotide (Rhod-Tz) **6** for immobilization (Fig. 2 *a*) (29). We chose to attach the labeled Tz to the immobilized DNA with the dienophile in solution to enable the observation of the quenched Rhod-Tz before, during, and after its reaction with the dienophile in single-molecule experiments. Rhod-Tz **6** shows very similar reactivity toward the dienophiles BCN and TCO as the other fluorogenic dye-Tz conjugates we tested; the reaction with BCN leads to a steady increase to a ca. threefold intensity increase after about 20 min, while the reaction with TCO shows a ca. 2.6-fold intensity peak after about 2 min, which decays to a ca. 1.1-fold intensity level over the following 2 h (Fig. S6).

Like in previously published protocols (35), we used streptavidin to immobilize a biotinylated complementary DNA template on a glass surface passivated with BSA and doped with biotinylated BSA. The DNA oligonucleotide was labeled with ATTO488 as reference for the immobilized samples. Subsequently, the fluorogenic Rhod-Tz **6** was hybridized to the immobilized DNA template. The DNA oligonucleotides were designed to ensure only negligible energy transfer between ATTO488 and the rhodamine moiety due to opposite orientations on the double-stranded DNA and large spacing. With the immobilized Rhod-Tz, we performed single-molecule fluorescence microscopy experiments in two-color TIRF mode. Surface density for immobilization of Rhod-Tz has been optimized by variation of concentration and incubation time of the various components to achieve sparse surface densities suitable for single-molecule detection (Fig. S7).

Single-molecule fluorescence emission intensities were automatically extracted and background corrected in adjacent and nonoverlapping positions in the same sample over the course of the reaction (see materials and methods and Fig. S1). This way, effects of photobleaching and photoinduced blinking are minimized, as light exposure is minimal for individual fluorophores and the addition of photostabilizing buffers potentially interfering with the reaction can be avoided. Although individual molecules are not observed while they undergo the reaction, single-molecule intensity distributions can be extracted that allow for subensemble discrimination of existing populations of different brightnesses.

The bulk reaction behavior of immobilized Rhod-Tz is confirmed by pseudoensemble intensity time courses generated from averaging single-molecule intensities per position plotted against the reaction time. Herein, the intensity time trace of Rhod-Tz upon reaction with TCO in Fig. 2 *b* shows the same characteristics as ensemble experiments with a steep initial rise shortly after TCO addition (marked with an asterisk [*]). In contrast to the reaction with BCN (Fig. S8), this is followed by a slow decay over the subsequent 3,000 s. This supports the assumption that any subpopulations seen in the single-molecule data reflect the occurrence of fluorescent species in the bulk reaction. For subensemble analysis, a two-dimensional time evolution of the single-molecule intensity distribution is shown in Fig. 2 *c*. The resulting time evolution of the single-molecule intensity distributions clearly illustrates that, initially, a bright population of molecules is formed rapidly after addition of TCO (at 900 s). Subsequently, this population shrinks again, while a simultaneous increase of the dark population is observed. About 50 min after the addition of TCO, the histogram closely resembles the initial distribution (see also Fig. S8 *a*).

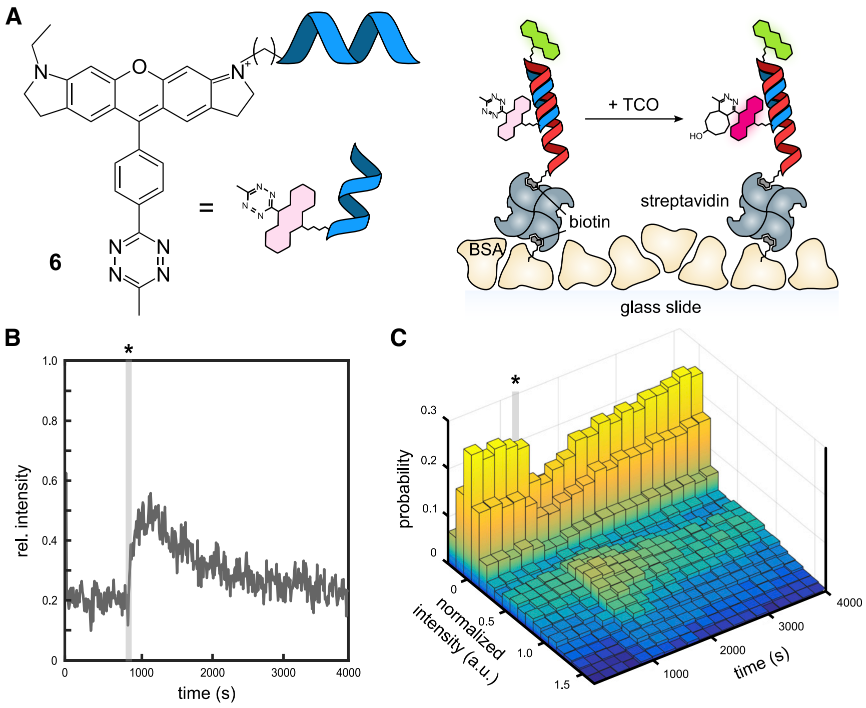

FIGURE 2 (*A*) The oligonucleotide coupled Rhod-Tz **6** and a schematic of its immobilization on glass by hybridization to immobilized complementary DNA oligonucleotide. The complementary DNA oligonucleotide was labeled with ATTO488 for reference and biotin for immobilization with streptavidin on BSA/BSA-biotin-coated glass surfaces. (*B*) Pseudoensemble time course of the reaction of immobilized Rhod-Tz under excitation at λ_{ex} 561 nm after the addition of TCO (marked with an asterisk [*]) by averaging over 400 frames from single-molecule experiments. (*C*) The three-dimensional visualization of the background-corrected and -normalized intensity distributions (200 s binning) evolving upon addition of TOC (marked with an asterisk [*]) show a rapid formation of a bright population centered at 0.7 a.u., which continuously returns to a nonfluorescent subpopulation.

Notably, single-molecule intensity distributions recorded over time show only two populations, namely a single low-intensity distribution centered at 0.0 a.u. and a single bright species centered at 0.7 a.u., without any sign of a species of intermediate brightness. This would be signified by a diagonal shift of the distribution mode or a broadening toward lower brightness. Moreover, the intensity of the bright population is comparable to the one observed for the reaction of immobilized Rhod-Tz with BCN (Fig. S8, *b*–*d*).

These results strongly suggest that only a single fluorescent species is present. Therefore, we conclude that upon reaction with TCO, the fluorogenic dye Rhod-Tz rapidly forms the fluorescent 4,5-dihydropyridazine **2**, which is converted over the subsequent 50 min to the respective 1,4-dihydropyridazine **3**. We identify **3** as a dark species with no or only very weak fluorescence emission. These findings are in good agreement with the UPLC-MS analysis (Fig. 1 *b*) as well as with the recent findings of Pinto-Pachecco et al. and the known reaction sequence (26,34).

DISCUSSION

The bioorthogonal reaction between Tzs and strained cyclic alkenes or alkynes offers a powerful tool for live-cell labeling. Beyond the high compatibility with living systems and fast kinetics, this reaction also features a fluorogenic effect if the used fluorophore is efficiently quenched by the Tz. Upon reaction with a dienophile, the quenching group is chemically transformed, resulting in an increase of fluorescence emission intensity.

The experimental reaction studies on fluorogenic Tzs presented in this work comprises a direct comparison of two commonly used dienophiles, BCN and TCO, with particularly fast reaction kinetics. Despite their structural relationship, they showed a fundamental difference in reactivity with fluorogenic Tzs. While a uniform increase of fluorescence toward saturation was observed with the alkyne BCN, the alkene TCO showed an initial increase followed by an intensity decay within minutes. Subsequently, another increase of fluorescence brightness can be observed over the course of several days or after the addition of oxidizing agents. The combination of UPLC-MS analysis with single-molecule fluorescence experiments allowed the assignment of the formed intermediates and improved our understanding of the underlying molecular processes. The reaction sequence from the initially formed 4,5-dihydropyridazine to the tautomeric 1,4-dihydropyridazine and oxidation to a pyridazine coincide with the observed fluorescence emission intensity time course. The fluorescence brightness of the oxidized TCO pyridazine product is comparable to the structurally similar pyridazine formed with BCN. While the brightness of the transiently forming intermediates could not be determined directly in ensemble experiments, single-molecule intensity distributions measured over the reaction course showed that the

emission intensity of 4,5-dihydropyridazine is also comparable to the BCN pyridazine. In contrast, the dark 1,4-dihydropyridazine exhibits significantly weaker fluorescence comparable to the quenched Tz-fluorophore conjugate prior to addition of the dienophile.

The peculiar reactivity of TCO with fluorogenic Tzs is of special importance in the context of probe development (36). While it is a common methodology to assess the fluorogenic effect of a Tz fluorophore by measuring the emission intensity increase upon reaction with a dienophile, it could be shown here that the achievable intensity increase largely depends on experimental conditions. The apparent ensemble brightness of the reaction mixture depends on the molecular composition of the different species, which changes over time due to secondary reactions. Thus, a meaningful and comparable value is hardly obtainable from these time course measurements. Furthermore, it could be shown that the peak intensity increase is subject to changes of the reaction speed of the initial step and herein depends on experimental settings, like temperature, concentration, and stoichiometry. As a consequence, intensity increases determined in this way do not represent the molecular brightness of a single reaction product nor do they allow an interpretation of the quenching efficiency of the Tz starting material. Therefore, more robust benchmarking of click reactions with Tz dyes and further advances in probe development are demanded to provide alternative dienophiles with fast kinetics and stable florescence emission. This will benefit fluorogenic labeling for different advanced fluorescence microscopy methods, including, but not limited to, quantitative and superresolution approaches.

SUPPORTING MATERIAL

Supporting material can be found online at https://doi.org/10.1016/j.bpr.2022.100084.

AUTHOR CONTRIBUTIONS

R.W. and D.-P.H. conceptualized the study. P.W. synthesized and provided the needed compounds. F.H. developed the microscope control with the help of K.Y. F.H. planned and conducted the experiments with the help of P.W., K.Y., R.W., and D.-P.H. F.H. and P.W. analyzed, plotted, and interpreted the data with the help of K.Y., R.W., and D.-P.H. D.-P.H. wrote the manuscript with the help of R.W., F.H., and P.W. All authors supervised and revised the manuscript.

ACKNOWLEDGMENTS

D.-P.H. and R.W. acknowledge funding from the Deutsche Forschungsgemeinschaft DFG (HE4559/6-1 and HE4559/5-1, SPP1623 WO1888/2-1). D.-P.H. also acknowledges funding by the Academy of Medical Sciences (APR2\1013). We thank Dr. Jürgen H. Gross (Heidelberg University) for support with UPLC-MS and Sebastian Intorp for helpful discussions.

DECLARATION OF INTERESTS

The authors declare no competing interests.

REFERENCES

1. Lang, K., and J. W. Chin. 2014. Bioorthogonal reactions for labeling proteins. *ACS Chem. Biol.* 9:16–20.
2. Plass, T., S. Milles, …, E. A. Lemke. 2011. Genetically encoded copper-free click chemistry. *Angew. Chem. Int. Ed. Engl.* 50:3878–3881.
3. Schoch, J., M. Wiessler, and A. Jäschke. 2010. Post-synthetic modification of DNA by inverse-electron-demand Diels–Alder reaction. *J. Am. Chem. Soc.* 132:8846–8847.
4. Ploschik, D., F. Rönicke, …, H. A. Wagenknecht. 2018. DNA primer extension with cyclopropenylated 7-Deaza-2′-deoxyadenosine and efficient bioorthogonal labeling in vitro and in living cells. *Chembiochem.* 19:1949–1953.
5. Agarwal, P., B. J. Beahm, …, C. R. Bertozzi. 2015. Systemic fluorescence imaging of zebrafish glycans with bioorthogonal chemistry. *Angew. Chem. Int. Ed. Engl.* 54:11504–11510.
6. Shieh, P., V. T. Dien, …, C. R. Bertozzi. 2015. CalFluors: a universal motif for fluorogenic azide probes across the visible spectrum. *J. Am. Chem. Soc.* 137:7145–7151.
7. Yang, J., J. Šečkutė, …, N. K. Devaraj. 2012. Live-cell imaging of cyclopropene tags with fluorogenic tetrazine cycloadditions. *Angew. Chem.* 124:7594–7597.
8. Neef, A. B., and C. Schultz. 2009. Selective fluorescence labeling of lipids in living cells. *Angew. Chem. Int. Ed. Engl.* 48:1498–1500.
9. Zheng, Q., and L. D. Lavis. 2017. Development of photostable fluorophores for molecular imaging. *Curr. Opin. Chem. Biol.* 39:32–38.
10. Wang, L., M. S. Frei, …, K. Johnsson. 2019. Small-molecule fluorescent probes for live-cell super-resolution microscopy. *J. Am. Chem. Soc.* 141:2770–2781.
11. Devaraj, N. K., R. Weissleder, and S. A. Hilderbrand. 2008. Tetrazine-based cycloadditions: application to pretargeted live cell imaging. *Bioconjug. Chem.* 19:2297–2299.
12. Devaraj, N. K., S. Hilderbrand, …, R. Weissleder. 2010. Bioorthogonal turn-on probes for imaging small molecules inside living cells. *Angew. Chem. Int. Ed. Engl.* 49:2869–2872.
13. Oliveira, B. L., Z. Guo, and G. J. L. Bernardes. 2017. Inverse electron demand Diels-Alder reactions in chemical biology. *Chem. Soc. Rev.* 46:4895–4950.
14. Reinkemeier, C. D., C. Koehler, …, E. A. Lemke. 2021. Synthesis and evaluation of novel ring-strained noncanonical amino acids for residue-specific bioorthogonal reactions in living cells. *Chem. Eur J.* 27:6094–6099.
15. Baalmann, M., L. Neises, …, R. Wombacher. 2020. A bioorthogonal click chemistry toolbox for targeted synthesis of branched and well-defined protein–protein conjugates. *Angew. Chem. Int. Ed. Engl.* 59:12885–12893.
16. Galeta, J., R. Dzijak, …, M. Vrabel. 2020. A systematic study of coumarin–tetrazine light-up probes for bioorthogonal fluorescence imaging. *Chemistry.* 26:9945–9953.
17. Németh, E., G. Knorr, …, P. Kele. 2020. A bioorthogonally applicable, fluorogenic, large Stokes-shift probe for intracellular super-resolution imaging of proteins. *Biomolecules.* 10:397.
18. Wieczorek, A., T. Buckup, and R. Wombacher. 2014. Rigid tetrazine fluorophore conjugates with fluorogenic properties in the

inverse electron demand Diels-Alder reaction. *Org. Biomol. Chem.* 12:4177–4185.

19. Lukinavičius, G., K. Umezawa, …, K. Johnsson. 2013. A near-infrared fluorophore for live-cell super-resolution microscopy of cellular proteins. *Nat. Chem.* 5:132–139.
20. Grimm, J. B., B. P. English, …, L. D. Lavis. 2015. A general method to improve fluorophores for live-cell and single-molecule microscopy. *Nat. Methods.* 12:244–250.
21. Wieczorek, A., P. Werther, …, R. Wombacher. 2017. Green- to far-red-emitting fluorogenic tetrazine probes - synthetic access and no-wash protein imaging inside living cells. *Chem. Sci.* 8:1506–1510.
22. Werther, P., K. Yserentant, …, R. Wombacher. 2021. Bio-orthogonal red and far-red fluorogenic probes for wash-free live-cell and super-resolution microscopy. *ACS Cent. Sci.* 7:1561–1571.
23. Werther, P., K. Yserentant, …, R. Wombacher. 2020. Live-cell localization microscopy with a fluorogenic and self-blinking tetrazine probe. *Angew. Chem. Int. Ed. Engl.* 59:804–810.
24. Egyed, A., A. Kormos, …, P. Kele. 2020. Bioothogonally applicable, π-extended rhodamines for super-resolution microscopy imaging for intracellular proteins. *Bioorg. Med. Chem.* 28, 115218.
25. Wang, L., M. Tran, …, K. Johnsson. 2020. A general strategy to develop cell permeable and fluorogenic probes for multicolour nanoscopy. *Nat. Chem.* 12:165–172.
26. Pinto-Pacheco, B., W. P. Carbery, …, D. Buccella. 2020. Fluorescence quenching effects of tetrazines and their Diels–Alder products: mechanistic insight toward fluorogenic efficiency. *Angew. Chem. Int. Ed. Engl.* 59:22140–22149.
27. Cordes, T., and S. A. Blum. 2013. Opportunities and challenges in single-molecule and single-particle fluorescence microscopy for mechanistic studies of chemical reactions. *Nat. Chem.* 5:993–999.
28. Rybina, A., C. Lang, …, D.-P. Herten. 2013. Distinguishing alternative reaction pathways by single-molecule fluorescence spectroscopy. *Angew. Chem. Int. Ed. Engl.* 52:6322–6325.
29. Werther, P., J. S. Möhler, and R. Wombacher. 2017. A bifunctional fluorogenic rhodamine probe for proximity-induced bioorthogonal chemistry. *Chemistry.* 23:18216–18224.
30. Edelstein, A., N. Amodaj, …, N. Stuurman. 2010. Computer control of microscopes using μManager. *Curr. Protoc. Mol. Biol.* 92:14.20.1–14.20.17.
31. Edelstein, A. D., M. A. Tsuchida, …, N. Stuurman. 2014. Advanced methods of microscope control using μManager software. *J. Biol. Methods.* 1:e10.
32. Schindelin, J., I. Arganda-Carreras, …, A. Cardona. 2012. Fiji: an open-source platform for biological-image analysis. *Nat. Methods.* 9:676–682.
33. Preus, S., L. L. Hildebrandt, and V. Birkedal. 2016. Optimal background estimators in single-molecule FRET microscopy. *Biophys. J.* 111:1278–1286.
34. Sauer, J., D. K. Heldmann, …, J. Schuster. 1998. 1, 2, 4, 5-tetrazine: synthesis and reactivity in [4+2] cycloadditions. *Eur. J. Org. Chem.* 1998:2885–2896.
35. Kiel, A., J. Kovacs, …, D.-P. Herten. 2007. Direct monitoring of formation and dissociation of individual metal complexes by single--molecule fluorescence spectroscopy. *Angew. Chem. Int. Ed. Engl.* 46:3363–3366.
36. Beliu, G., A. J. Kurz, …, M. Sauer. 2019. Bioorthogonal labeling with tetrazine-dyes for super-resolution microscopy. *Commun. Biol.* 2:261.

Biophysical Reports, Volume *2*

Supplemental information

A dark intermediate in the fluorogenic reaction between tetrazine fluorophores and *trans*-cyclooctene

Felix Hild, Philipp Werther, Klaus Yserentant, Richard Wombacher, and Dirk-Peter Herten

Supplemental Information

A dark intermediate in the fluorogenic reaction between tetrazine fluorophores and trans-cyclooctene

Felix Hild, Philipp Werther*, Klaus Yserentant, Richard Wombacher, Dirk-Peter Herten*

* These authors contributed equally to the manuscript.

Figure S1

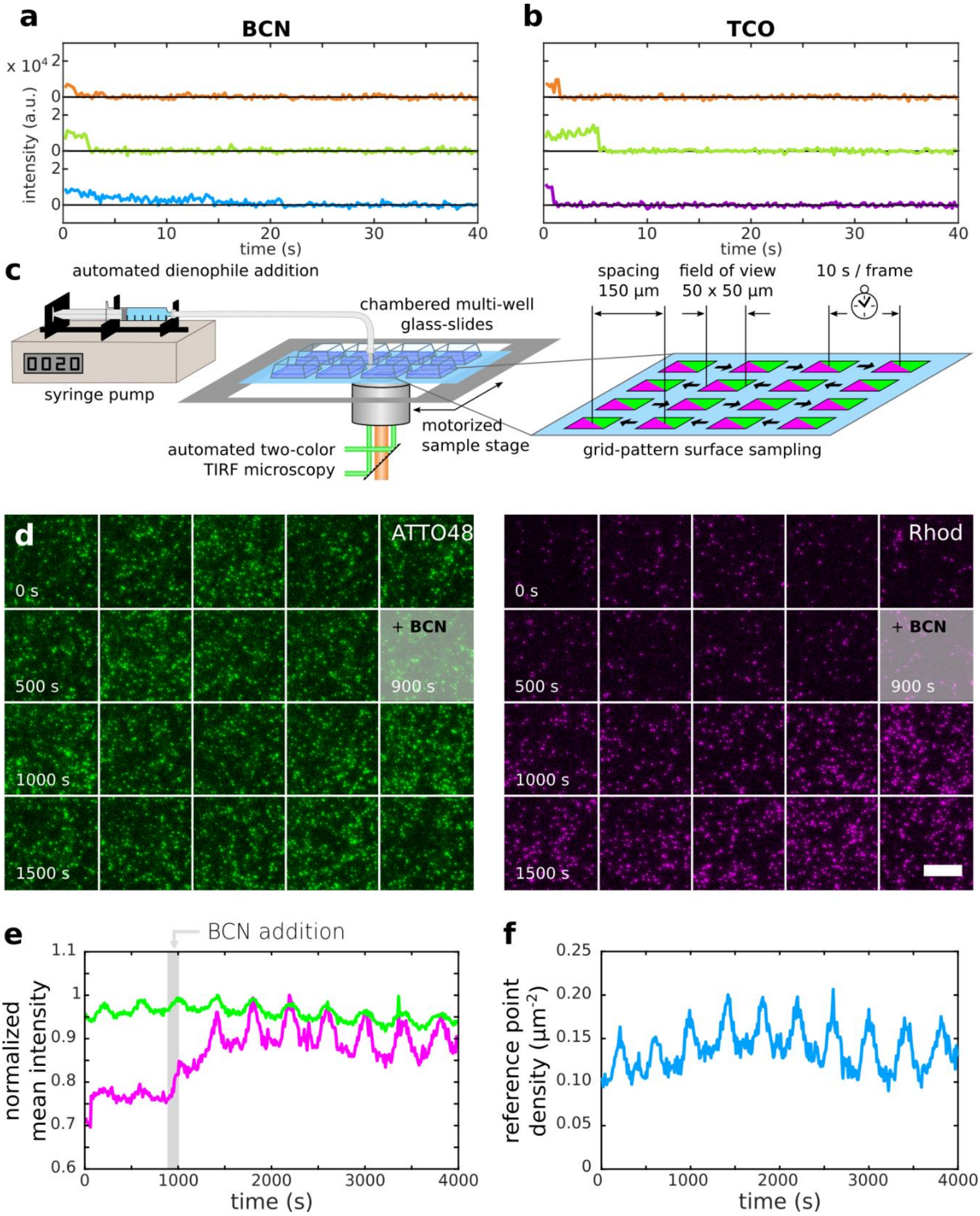

Figure S1: Examples of single molecule intensity traces of IEDDA reaction products of immobilised Rhod-Tz reacted with 50 µM a) BCN and b) TCO. The rapid photo-bleaching of these products required a different concept of time-resolved subpopulation probing explained in the following. c) Automated two-colour time-lapse single-molecule microscopy of fluorogenic IEDDA reaction was realized with automated dienophile addition with a motorized syringe pump, a motorized stage moving chambered glass slides and an automated multi-colour microscopy acquisition. To sample the progress of the reaction the glass surface was scanned at 10 s / frame in a serpentine pattern with 150 µm spacing. d) Microscopy images show an increase in point density in the Rhod channel (magenta) after BCN addition at nearly constant density in the ATTO488 reference channel (green). e) Normalized mean image intensity profiles show an increase after addition in the magenta channel with superimposed periodic fluctuations in both channels due to variations in reference point density (f). Scale bar 10 µm. Snapshots of acquired images show a roughly constant point density for ATTO488 in (d). In contrast, the point density of Rhod-Tz increases significantly after addition of BCN at 900 s. This effect is clearly visible in a plot of mean image intensity normalized to the maximum of the sequence (e). Additionally, it shows periodic fluctuations correlating between the two spectral channels. These variations occurring with a spacing of 20 frames are due to a variation of template density over the scanned region which controls the achievable density of Rhod and is reflected in the mean intensity of the images. This is evident with a plot of the density of points detected in theATTO488 channel over time in (f). Such large-scale inhomogeneities are frequently observed for fluorophores immobilized by this concept and could be a result of uneven surface quality of the glass or non-uniform treatment during sample preparation. Generally, this should not impose a problem as the observed densities of 0.1–0.2 µm-2 correspond to 250–500 points per field of view and herein provides sufficient data for statistically relevant analysis.

Figure S2

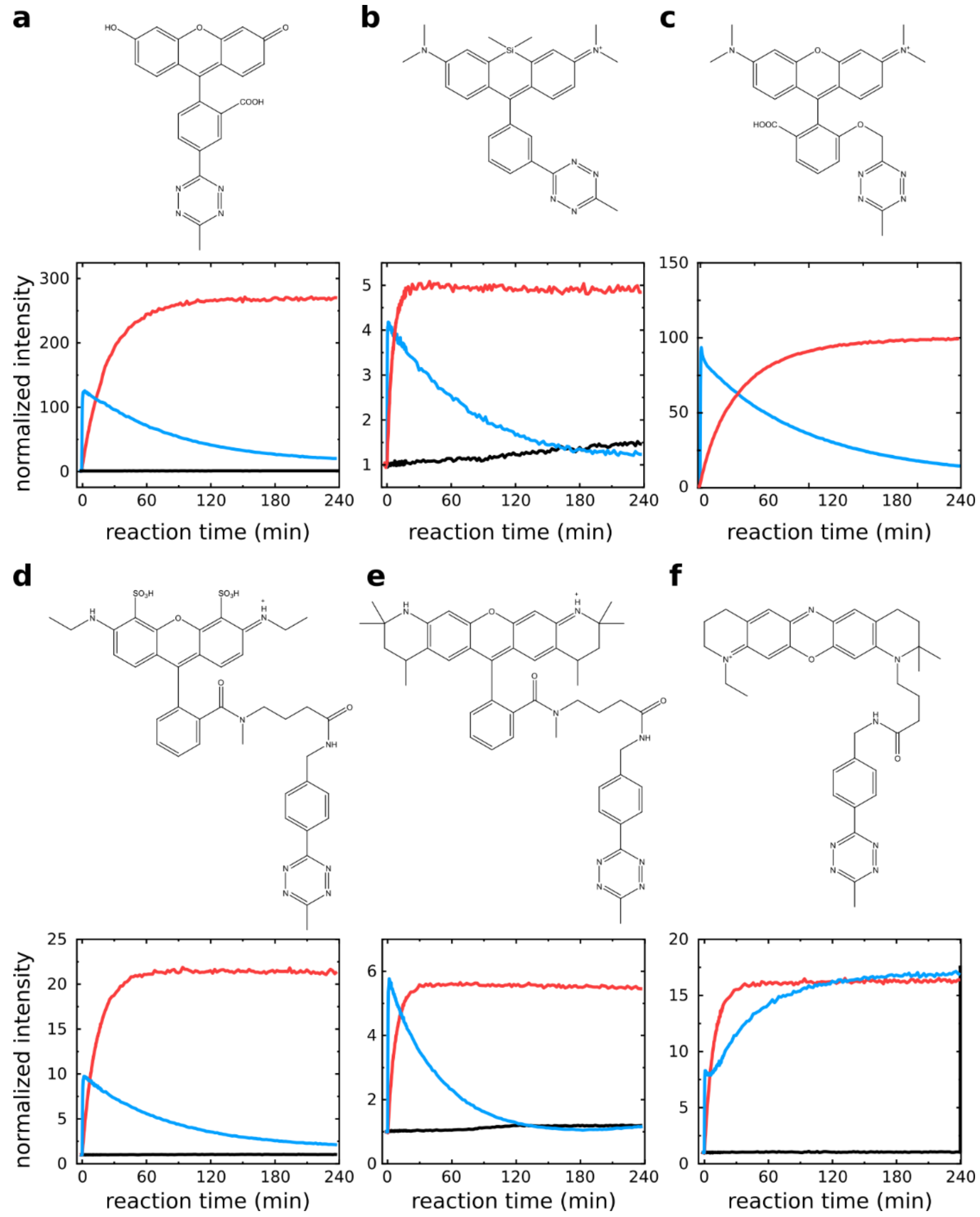

Figure S2: Fluorescence time course measurements of fluorogenic Tz dyes. a) Fl-Tz, b) SiR-Tz, c) TMR-Tz, d) ATTO532-Tz, e) ATTO550-Tz and f) ATTO655-Tz, all 5 µM in PBS, reacting with a 20-fold excess of BCN (red) or TCO (blue). Fluorescence intensity was recorded over 4 h at the wavelengths of the individual emission maxima. All dyes reacted with TCO except ATTO655-Tz show a significant drop in intensity after about 5 mins. ATTO655-Tz reproducibly shows a steep increase with only a short and small decrease to an intermediate level within 5 mins followed by an increase over 2 h saturating at an intensity that is comparable to the reaction with BCN. This is most likely due to a faster oxidation to the final pyridazine. Control samples (black) show neglectable increase for a), b), d), e) and f) and a higher instability for c).

Figure S3

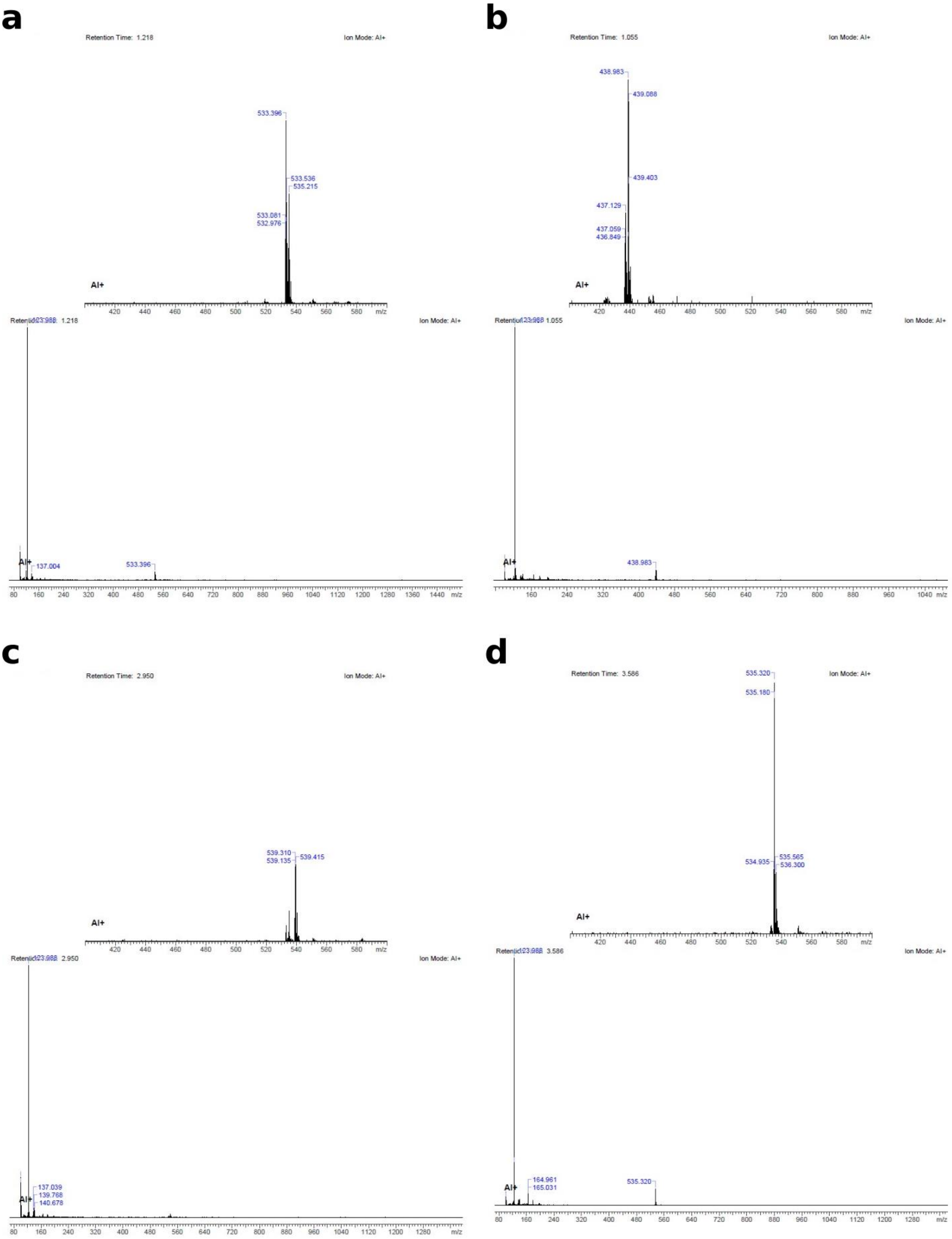

Figure S3: Mass spectra of relevant peaks from the ULPC-MS experiments in fig. 1b. a) Peak I at t = 14d, b) peak II at t = 8d, c) peak IV at t = 5 min and d) peak V at t = 8h.

Figure S4

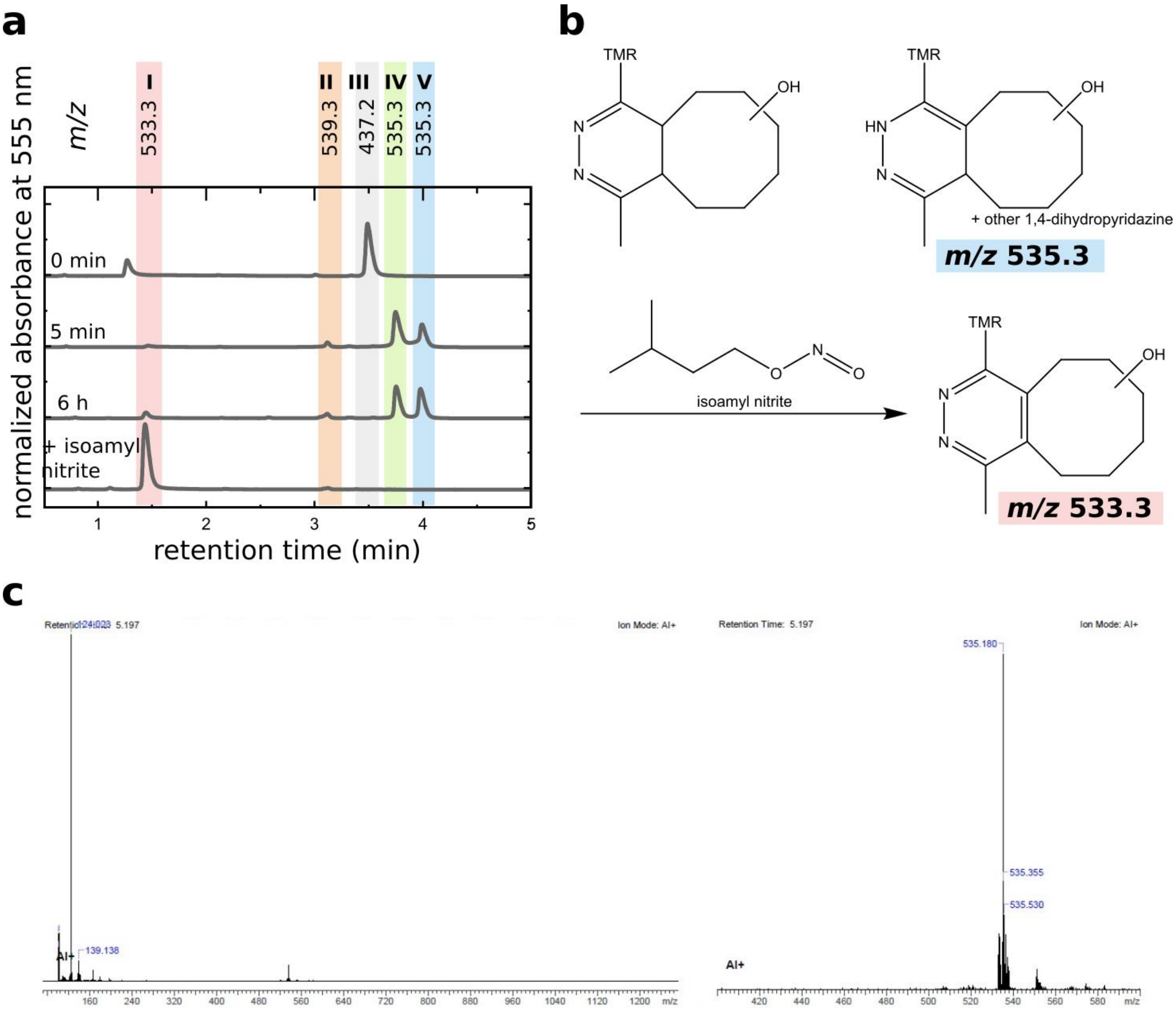

Figure S4: a) UPLC-MS analysis of the b) oxidation of dihydropyridazines with isoamyl nitrite. 4,5- and 1,4-dihydropyridazines (peaks IV and V) were oxidized by addition of 100 eq of isoamyl nitrite yielding a single peak with m/z 533.3 corresponding to the pyridazine (peak I). c) Mass spectrum of peak I after oxidation with isoamylnitride.

Figure S5

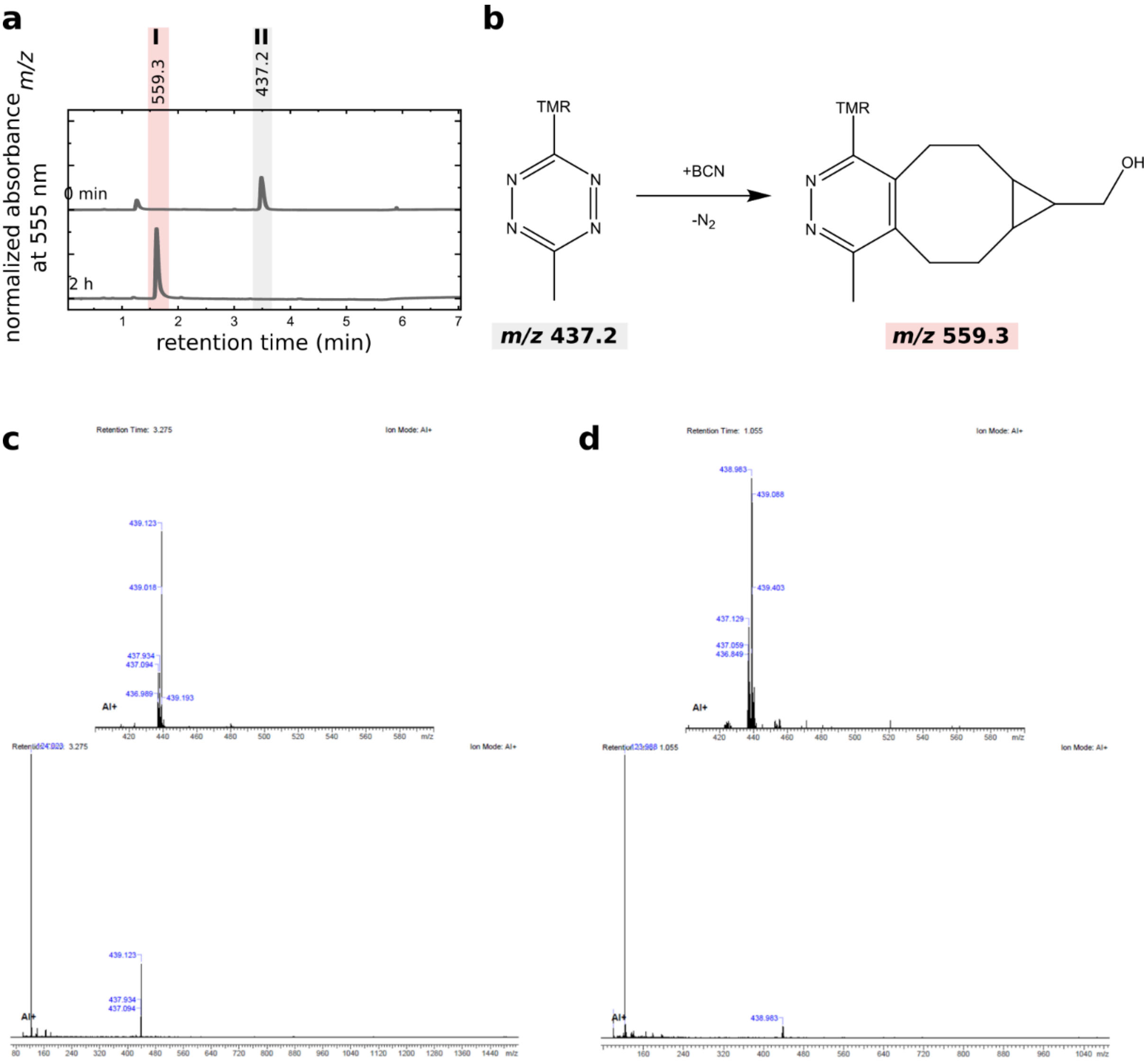

Figure S5: UPLC-MS analysis of TMR-Tz reacting with BCN. a) Chromatograms of the b) reaction of TMR-Tz (m/z 437.2) at 200 µM in PBS before (0 min) and 2 h after addition of 20 equivalents of BCN to form a single pyridazine product (m/z 559.3). c) Mass spectra of peak II at t = 0 and d) peak I at t = 2h.

Figure S6

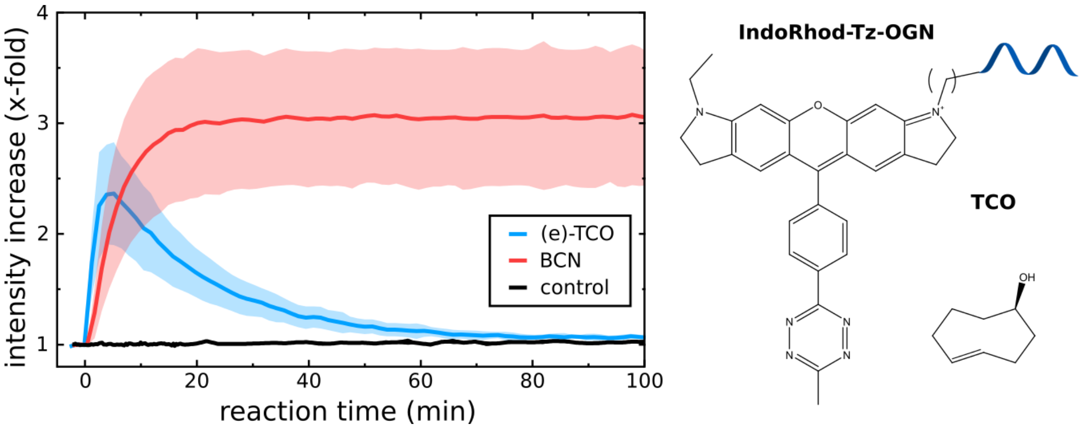

Figure S6: Reaction of the oligonucleotide coupled IndoRhod-Tz with dienophiles. Time course measurements of fluorescence intensity at λ_{em} 595 nm excited at λ_{ecx} 535 nm. Reaction with TCO (blue) yields a steep increase followed by a decrease nearly down to the initial intensity level. BCN (red) shows a slower increase to a constant level. Control samples (black) without dienophile addition. Lines represent the average of three measurements with the standard deviation shown as transparent bands.

Figure S7

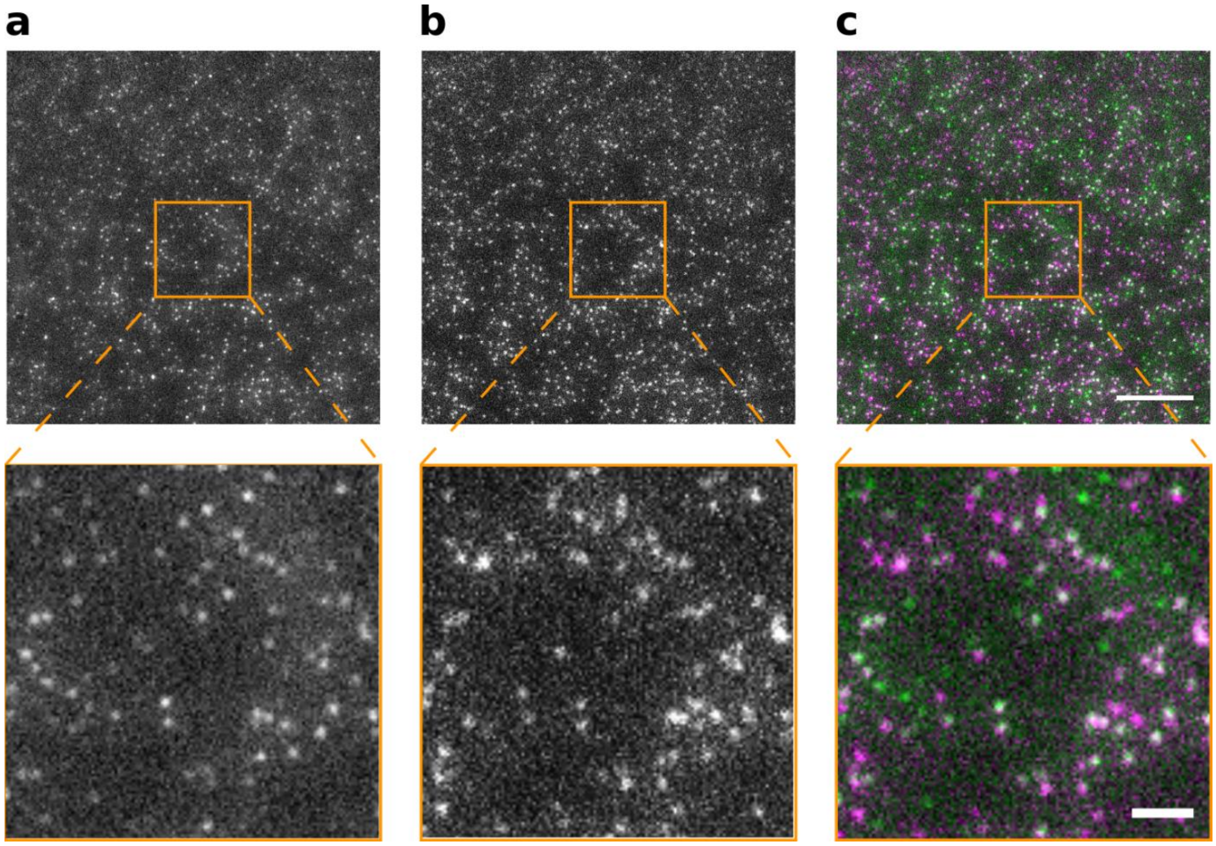

Figure S7: Two-colour TIRF microscopy of immobilized Rhod-Tz after reaction with BCN. The template oligonucleotide labelled with ATTO488 (green) and Rhod-Tz after reaction with BCN (magenta) shows a high degree of colocalization (white points in overlay). Scale bar 10 µm (top) and 2 µm (bottom).

Figure S8

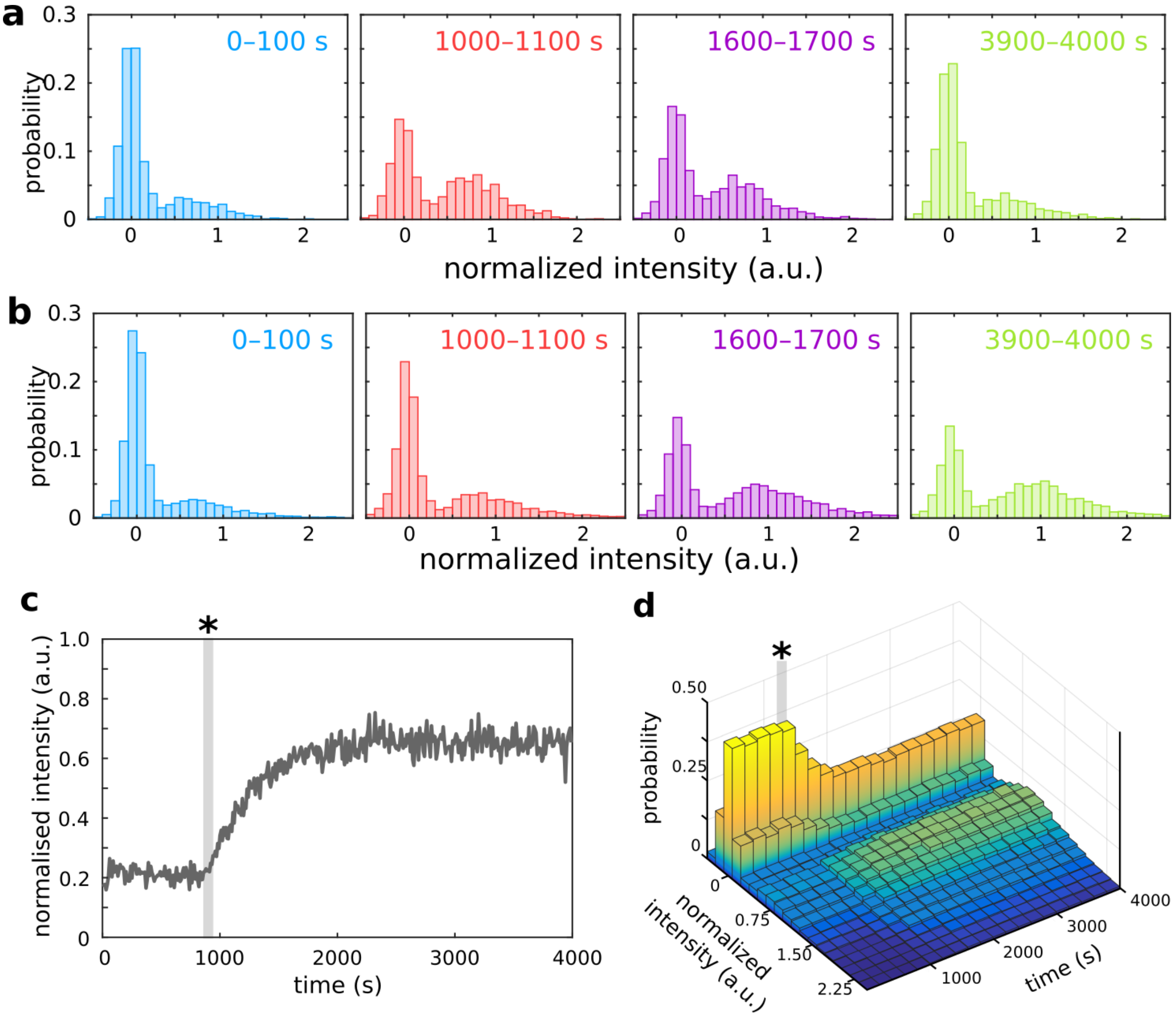

Figure S8: Time-resolved single molecule studies of the reaction of Rhod-Tz with different dienophiles. a) Intensity distributions after 100, 1100, 1700 and 4000 s show the formation of a bright population briefly after TCO addition at 900 s while the background population simultaneously decreases. Over time the trend is reversed and results in a distribution after ca. 4000 s that approximately corresponds to the initial distribution. b) Intensity histograms after 100, 1100, 1700 and 4000 s show a steady increase of a bright population after addition of BCN (added at 900 s) while the background intensity population decreases. c) Ensemble intensity time course of the reaction of Rhod-Tz after addition of BCN (marked with *) from averaging intensities per frame (grey, top) shows the corresponding overall intensity. d) The 3D-visualization of background corrected and normalized intensity distributions upon addition of BCN (marked with *) plotted against time shows a continuous growth of the bright population along with a simultaneous decrease of the dark population saturating about 1000 s after BCN addition.
